# Supplementary material for: Lifestyle advice to cancer survivors: a qualitative study on the perspectives of health professionals
Source: BMJ Open. 2018 Mar 27;8(3):e020313. doi: 10.1136/bmjopen-2017-020313 (PMC5875617; doi:10.1136/bmjopen-2017-020313)
Supplement: Supplementary file 1 [file bmjopen-2017-020313supp001.pdf]

Supplementary material 1: Interview Topic Guide

	Topic	Question	Prompt
1.	Introductions / background	Brief introductions; get verbal consent to tape-record the interview; remind the aims of study, check length of interview/audio-recorded.	
2.	Health beliefs	What are your views on the role of diet and physical activity in the recovery from [breast/prostate/colorectal] cancer?<br>What are your views on the role of diet and physical activity in cancer recurrence?	
3.	Awareness of lifestyle guidelines for cancer survivors	Are you familiar with any guidelines specifically for cancer patients for any of the following lifestyle topics?<br>You said that you were aware of "X, Y, Z" guidelines. Can you please tell me what you know (or what you remember) about each of these guidelines?	Prompt<br>- PA<br>- Diet<br>- Weight management<br>- Smoking<br>- Alcohol
		What are your views on these guidelines? What do you think about them?	Prompt<br>- Liking<br>- Realistic<br>- Reliable
		How did you find out about them?<br>You also said that you were not aware of the "K, M" guidelines. Why do you think that you did not come across these guidelines?<br>What were the barriers for you not getting this information?	
3.	Barriers for provision of lifestyle advice	Thinking of all your patients who have completed primary treatment for cancer, do you give your patients advice on any lifestyle topics?	Prompt<br>- PA<br>- Diet<br>- Weight management<br>- Smoking<br>- Alcohol
		You said that you give advice on "X lifestyle topic" to "X%" of your patients. What kind of advice do you tend to give?	Prompt<br>- Verbal

Topic	Question	Prompt
		- Written<br>- Website referral<br>- HP referral<br>- When / Why this specific format<br>- Guidelines based
	Why do you think that you provide advice on this to [so many / only to x%] of your patients?<br>What prevents you from providing this type of advice to more patients?	
	What are the reasons why you don’t usually provide lifestyle advice?	Prompt<br>- Verbal<br>- Written<br>- Website referral<br>- HP referral
	For each lifestyle topic that professional does NOT provide any advice, ask: Is there any particular reason why you have not given advice on “X lifestyle topic” to any of your patients?	
	What are the barriers preventing you to provide this advice?	Prompt<br>- Guidelines
	On the survey, we asked you about a number of specific barriers preventing you to provide lifestyle advice for your patients. You mentioned that “X“ and “Y” were the main barriers for you. Can you tell me more about these barriers? Why these prevent you from providing lifestyle advice?<br>Are there any other barriers?	
5. Content Expectations	Have any of your cancer patients ask you information about lifestyle?<br><br>What type of information do they usually ask for?<br>How often do they ask you about lifestyle information? Why?<br>Is there any other type of information they should be getting? Why do you think it is important they get that information?<br>Is there anything you would avoid in telling them? Why?	

Topic	Question	Prompt
	Have any of the relatives, family members, friends or carers of your patients asked you information about lifestyle?	
	What type of information do they usually ask for?	
	How often do they ask you about lifestyle information? Why?	
	Is there any other type of information they should be getting? Why do you think it is important they get that information?	
	Is there anything you would avoid in telling relatives, friends or carers? Why?	
6. Delivery	We are planning to develop some materials to provide information on diet and physical activity for people who have had cancer treatment.	
	When would it be the best time for health professionals to provide information on healthy eating and physical activity to colorectal cancer patients? Why?	
	Do you think patients would be open to receive this type of information at that time? Why?	
	Who should provide them information about healthy eating and physical activity? Why?	
	In what format do you think that it would be better to provide information about diet and physical activity, for colorectal cancer patients? Why?	
	What do you think about a leaflet?	Prompt
	What do you think about a leaflet along with a telephone call from a HP?	- Usefulness
	What do you think about a leaflet along with a face-to-face contact with a HP?	- Receipt
	What do you think about a leaflet along with online information?	- Implementation
	What do you think about a self-help book with specific information for cancer survivors?	
	What do you think about a group intervention designed for cancer survivors with about 8 sessions, providing more detailed lifestyle information?	
	We have discussed about several options and formats to provide lifestyle information. Balancing between what would be better for HPs, for patients and feasibility of implementation; what do you think would be the best format to provide lifestyle advice?	

	Topic	Question	Prompt
		From your perspective, what are the issues or barriers to take into account that some [breast/prostate/colorectal] cancer patients might face in trying to change their lifestyle?	Prompt<br>- Mood<br>- Mental health<br>- Physical restrictions<br>- Visual problems
7.	Ending	Do you have anything else to add, any comments or any questions?	
