# Supplementary material for: Lifestyle advice to cancer survivors: a qualitative study on the perspectives of health professionals
Source: BMJ Open. 2018 Mar 27;8(3):e020313. doi: 10.1136/bmjopen-2017-020313 (PMC5875617; doi:10.1136/bmjopen-2017-020313)
Supplement: Supplementary file 2 [file bmjopen-2017-020313supp002.pdf]

Supplementary material 2 – Interviews NVIVO Coding Framework

(1) Health Beliefs

- Beliefs about role of diet in cancer (incl. cancer recurrence)
- Beliefs about role of exercise in cancer (incl. cancer recurrence)
- Beliefs about role of alcohol in cancer (incl. cancer recurrence)
- Beliefs about role of smoking in cancer (incl. cancer recurrence)
- Health beliefs (not lifestyle)

(2) Awareness of lifestyle guidelines for cancer survivors

- Aware of guidelines (& description)
- Not aware of guidelines (& description)
- Views on guidelines
- Barriers to accessing guidelines (incl. not aware)
- How found out about guidelines

(3) Provision of lifestyle advice

- Advice given (diet)
- Advice given (exercise)
- Advice given (smoking)
- Advice given (alcohol)
- Advice given (weight)
- Advice given and requested by Relatives
- Advice would like to get on other topics
- Barriers to provision of advice
- Reason for provision of advice to a HIGH number of pts
- Reason for provision of advice to a LOW number of pts

(4) Format

- Timing
    - Timing HP has provided lifestyle advice
    - Preferred timing for provision of lifestyle advice
- Source

Mode of delivery (all negative, incl. leaflet, self-help book and group)

Ideal Mode of delivery

Mode of delivery of information provided (incl verbally, referral, web, leaflet, self help and group)

Format Group

Format Verbally

Format Digital

Format Written (All)

Leaflet only

Leaflet + telephone call

Leaflet + face-to-face contact

Leaflet + online information

Self-Help book

(5) Barriers to attending lifestyle interventions - internal & external

(6) Pathway issues

Leaflet only

Leaflet + telephone call

Leaflet + face to face contact

Leaflet + online information

Self-Help Book

Group intervention

Other Pathway factors
