# Supplementary material for: Lifestyle advice to cancer survivors: a qualitative study on the perspectives of health professionals
Source: BMJ Open. 2018 Mar 27;8(3):e020313. doi: 10.1136/bmjopen-2017-020313 (PMC5875617; doi:10.1136/bmjopen-2017-020313)
Supplement: Author's manuscript [file bmjopen-2017-020313.draft_revisions.pdf]

BMJ Open is committed to open peer review. As part of this commitment we make the peer review history of every article we publish publicly available.

When an article is published we post the peer reviewers' comments and the authors' responses online. We also post the versions of the paper that were used during peer review. These are the versions that the peer review comments apply to.

The versions of the paper that follow are the versions that were submitted during the peer review process. They are not the versions of record or the final published versions. They should not be cited or distributed as the published version of this manuscript.

BMJ Open is an open access journal and the full, final, typeset and author-corrected version of record of the manuscript is available on our site with no access controls, subscription charges or pay-per-view fees (http://bmjopen.bmj.com).

If you have any questions on BMJ Open's open peer review process please email info.bmjopen@bmj.com

Lifestyle advice to cancer survivors: a qualitative study on the perspectives of health professionals

Journal:	*BMJ Open*
Manuscript ID	bmjopen-2017-020313
Article Type:	Research
Date Submitted by the Author:	27-Oct-2017
Complete List of Authors:	Koutoukidis, Dimitrios; University College London, Behavioural Science and Health<br>Lopes, Sonia; University College London, Behavioural Science and Health<br>Fisher, Abigail; University College London, Behavioural Science and Health<br>Williams, Kate; University College London, Behavioural Science and Health<br>Croker, Helen; University College London, Behavioural Science and Health<br>Beeken, Rebecca; University College London, Behavioural Science and Health; University of Leeds Leeds Institute of Health Sciences
**Primary Subject Heading**:	Oncology
Secondary Subject Heading:	Qualitative research
Keywords:	health professionals, cancer survivors, lifestyle, guidelines, interventions

SCHOLARONE™ Manuscripts

For peer review only

Lifestyle advice to cancer survivors: a qualitative study on the perspectives of health professionals

Dimitrios A. Koutoukidis[1¶], Sonia Lopes[1¶], Abigail Fisher[1], Kate Williams[1], Helen Croker[1], Rebecca J. Beeken[1,2]

[1]Department of Behavioural Science and Health, University College London, London, UK

[2]Leeds Institute of Health Sciences, University of Leeds, Leeds, UK

[¶]Contributed equally

Corresponding Author:

Dr Rebecca Beeken

Leeds Institute of Health Sciences

Level 10, Worsley Building,

Clarendon Way,

Leeds, LS2 9NL Tel. +44 (0)113 343 3228 E-mail: r.beeken@leeds.ac.uk

For peer review only - http://bmjopen.bmj.com/site/about/guidelines.xhtml

Abstract

Objectives: Adoption of healthy lifestyle behaviours has shown promising effectiveness in reducing the high morbidity burden of cancer survivors. Health professionals (HPs) are well suited to provide lifestyle advice but few survivors report receiving guidance from them. The study aimed to explore HPs' perspective of lifestyle advice (on heathy eating, physical activity, smoking, and alcohol) for cancer survivors.

Design: In-depth semistructured qualitative interviews were conducted by telephone or face-to-face. Data were analysed using qualitative content analysis.

Setting and participants: Twenty-one UK HPs working in secondary care with breast, prostate, or colorectal cancer survivors were interviewed.

Results: The over-arching theme was that HPs' desire to provide lifestyle advice was not necessarily matched by knowledge and action. Three main themes were identified: 1) survivorship-centred barriers to provision, 2) clinician-centred barriers to provision, and 3) optimal delivery of lifestyle advice. Results suggested that survivors' current status of practising health behaviours, their perceived socio-economic barriers and ability to practise health behaviours, and HPs' fear for potential loss of connection with the patient influenced provision of lifestyle advice. Further factors included clinicians' knowledge of healthy lifestyle guidelines, feeling that they were not the 'right person' to provide advice, and lack of time and resources. HPs stressed that the optimal delivery of lifestyle advice should (a) be tailored to the individual and delivered throughout the cancer journey, (b) be focused on small and achievable changes framed as part of their treatment regimen, and (c) be cost-effective for wide-scale implementation.

Conclusions: Incorporation of the identified barriers when developing HP training programmes and lifestyle interventions could increase the probability of successful behaviour change and, thus, improved outcomes for cancer survivors.

Keywords: health professionals, cancer survivors, lifestyle, guidelines, interventions

For peer review only - http://bmjopen.bmj.com/site/about/guidelines.xhtml

For peer review only

Strengths and limitations of the study

- The study comprehensively examined the views of health professionals, working with breast, prostate, and colorectal cancer survivors, on all major lifestyle topics.
- Health professionals had diverse professional backgrounds, including nursing, oncology, surgery, and allied health professions.
- It is possible that the participants were generally interested in lifestyle topics, which might bias the responses towards a positive view on lifestyle.
- Although qualitative studies provide in-depth information, their findings cannot be generalised.

For peer review only

For peer review only - http://bmjopen.bmj.com/site/about/guidelines.xhtml

Introduction

There are 2.5 million cancer survivors living in the UK.[1] The increasing number of cancer survivors and their higher morbidity burden compared to the general population means strategies are needed to improve survivorship outcomes.[2 3] Adoption of healthy behaviours, such as not smoking or drinking alcohol, eating a healthy diet, engaging in sufficient physical activity, and weight management, are potential strategies. There is a growing body of evidence that suggests these health behaviours are positively associated with improved outcomes in cancer survivors.[4 5] Furthermore, behavioural lifestyle interventions in this population have shown promising effectiveness.[6 7]

There are now lifestyle guidelines for cancer survivors, advocating smoking cessation, moderate alcohol consumption (if any), a healthy balanced diet, daily physical activity, and maintenance of a healthy weight.[8-11] Although surveys suggest that few cancer survivors are meeting these guidelines,[12] qualitative data suggest that a cancer diagnosis may promote motivation for behaviour change and that survivors are keen to receive healthy lifestyle advice, ideally from a credible source.[13 14] Health professionals (HPs) are well positioned to provide healthy lifestyle advice and, thus, help cancer survivors improve their lifestyle behaviours. However, few survivors report receiving lifestyle advice from their HPs.[13 15]

We conducted a survey suggesting that 36% of HPs were not aware of any lifestyle guidelines.[16] Although most reported providing some lifestyle advice, a number of barriers to provision were also cited, including lack of guidelines, belief that advice would not change behaviour or affect outcomes, belief that they are not the right person to provide advice, lack of time, lack of patient interest, belief that they would blame the patient, and cultural differences. These highlighted the potential for an intervention to help HPs provide evidence-based advice.

This qualitative study sought to build on this work by exploring in greater depth HPs' understanding of cancer and lifestyle and the related guidelines, as well as the advice HPs are giving to survivors when knowledge of guidelines is lacking. Furthermore, it aimed to identify barriers to providing advice, and preferences for interventions to facilitate the provision of advice in the oncology setting.

For peer review only - http://bmjopen.bmj.com/site/about/guidelines.xhtml

Methods

Procedure and Participants

Eligible participants were UK-based HPs (including surgeons, physicians, nurses and allied HPs) caring for patients diagnosed with breast, prostate, or colorectal cancer. Following participation in an online survey on lifestyle guidelines for cancer survivors,[16] HPs were invited via e-mail to participate in either a telephone (n=20) or an individual face-to-face (n=1) interview at University College London. Participants provided signed informed consent. Ethical approval was granted by the UCL Research Ethics Committee (project ID: 4456/001).

Interviews

Between October 2014 and January 2015, an experienced female research psychologist with interest in cancer survivorship (SL, MSc) interviewed the HPs. The interviewer had no previous relationship with the study participants. Participants were aware that the study was part of the interviewer's research degree. Each interview lasted approximately 40 minutes (range: 17-74 minutes). The interview followed a semi-structured guide (Supplementary material 1). The guide followed up on HPs' individual survey responses about the provision of advice, and used open-ended questions and prompts to explore in-depth HPs' awareness of survivorship lifestyle guidelines, their barriers to provision of lifestyle advice, and their views on various formats for lifestyle interventions. Interviews were audio-recorded without field notes, anonymised, and transcribed verbatim. The researcher listened to the tapes to verify them for accuracy.

Analysis

Interview transcripts were analysed using qualitative content analysis[17] in NVivo version 10. Following familiarisation with the data, meaning units and codes were generated using an inductive approach. SL coded the transcripts derived the coding list (Supplementary material 2) in an iterative process with RJB. An independent reviewer with a dietetics background (DAK) coded 20% of interviews against the coding list. Inter-rater reliability was high (mean kappa=0.92) and minor coding differences were resolved by discussion. Categories and themes were compiled with a manifest analysis using an iterative process. This ensured internal homogeneity

For peer review only - http://bmjopen.bmj.com/site/about/guidelines.xhtml

115 and external heterogeneity of the themes and categories. The completed COREQ checklist on standard reporting guidelines is available in Supplementary material 3.[18]

117 ## Results

118 ### Sample Characteristics

119 Fifty-two of the 336 survey participants were eligible for this study. Twenty-one (40.4%) were interviewed and 31 either did not respond or were unwilling to take part. Table 1 shows the sample characteristics of the study in relation to the survey. Most participants were female and aged between 46 and 55 years. Their professional role varied and their work location spanned across the UK.

124 **Table 1:** Sample characteristics in relation to the previous survey

	Interview participants n=21	Survey participants n=336[1]
	% (n)	% (n)
Gender		
Female	76 (16)	81 (272)
Male	24 (5)	19 (64)
Age *(in years)*		
26-35	5 (1)	12 (41)
36-45	38 (8)	33 (110)
46-55	48 (10)	42 (141)
56-65	10 (2)	13 (42)
Profession		
Nurse	43 (9)	55 (126)
Physician	33 (7)	21 (48)
Surgeon	19 (4)	13 (31)
Allied HP (physiotherapist)	5 (1)	11 (26)
Cancer Specialism		
Breast	43 (9)	25 (54)
Colorectal	38 (8)	15 (32)
Prostate	19 (4)	10 (21)
Region of Work		
North West England	38 (8)	-
London	24 (5)	-
South West England	10 (2)	-
South East England	10 (2)	-
Wales	10 (2)	-
East of England	5 (1)	-

For peer review only - http://bmjopen.bmj.com/site/about/guidelines.xhtml

Scotland	5 (1)	-

[1] Data from Williams et al 2015. % may not total 100% due to rounding.

Themes

The overarching theme arising from the interviews was that HPs' desire to provide lifestyle advice was not necessarily matched by knowledge and action. Three main themes were identified: 1) survivorship-centred barriers to provision of lifestyle advice, 2) clinician-centred barriers to provision of lifestyle advice, and 3) optimal delivery of lifestyle advice.

1 Survivor-centred barriers to provision of lifestyle advice

1.1. Perceptions of survivors' current health behaviours

HPs felt that lifestyle behaviour change was particularly challenging for cancer survivors, due to ingrained lifelong habits and sustainability of achieved changes.

> *Well, lifestyle is very much a matter of habit, and breaking habits is always difficult for all of us.*
>
> Breast Cancer Surgeon, M, 63y

Co-existence of multiple suboptimal behaviours was regarded as a barrier to provision of lifestyle advice.

> *It is sometimes difficult when people just do seem to have all the vices, so they are heavy smokers, they are not just overweight, they are really obese, they obviously take no exercise and they are heavy smokers. It's very difficult to know sometimes where to start.*
>
> Breast Cancer Surgeon, F, 53y

Alcohol and tobacco were regarded as sensitive topics and it was perceived hard to assess consumption levels. Therefore, this was only addressed if there were evident indications of a problem or if the topic was raised by relatives.

> *[…] when it comes to alcohol it can be quite tricky. Sometimes we'll have relatives who will say, 'Are you aware this person is drinking?' particularly if they are on chemotherapy. And the side effects of chemotherapy, and then to add alcohol onto it. I think that's a more difficult area to try and challenge people on.*
>
> Colorectal Cancer Nurse, F, 54y

For peer review only

For peer review only - http://bmjopen.bmj.com/site/about/guidelines.xhtml

1.2 Perceived ability to perform health behaviours

The lifestyle advice provided was mostly tailored to the individual patient. Frailty and physical inability to perform physical activity significantly hindered the HPs' inclination to provide physical activity advice. Tolerability of a high-fibre diet due to treatment-related bowel changes was also considered a barrier for provision of advice on healthy eating. HPs also highlighted that lifestyle advice would be inappropriate for end-of-life survivors.

> *If somebody is frail and unwell generally, either because of their cancer or because of other medical problems, then I think advising them about diet and exercise and things isn't necessarily helpful for them.*
>
> Prostate Cancer Physician, F, 34y

They felt that low mood and distress were also barriers for implementation of the provided advice.

> *If someone is depressed or has low mood, for whatever reason, everything is harder.*
>
> Breast Cancer Nurse, F, 40y

1.3 Potential loss of connection with the patient

Fear of blaming the patient resulted in HPs taking a subtle rather than a strong approach to providing lifestyle advice. Clinicians did not want to make people feel guilty about their previous lifestyle or even create future blame if the patient did not subsequently change their lifestyle and the cancer reoccurred. They mentioned a potential loss of credibility and connection with their patients if they were to insist on lifestyle change.

> *I need to keep credibility with them as well, and if I start making demands of them which they really can't possibly keep then I kind of lose my own connection with these people.*
>
> Breast Cancer Surgeon, M, 63y

Another barrier was the obstruction of patients' freewill, as they believed people have their own views about what is healthy or unhealthy. Some felt that they should only start such a conversation if the patient were to bring up the topic. HPs perceived lifestyle to be a low priority for the majority of survivors, although they acknowledged the cancer diagnosis as a "teachable moment" for them.

For peer review only - http://bmjopen.bmj.com/site/about/guidelines.xhtml

I mean, a smoker, if they are determined not to give up there's absolutely nothing you can do about it. I mean, for some it's a real wake-up call and for some they really change their lifestyle. […] and then also sometimes people don't want to talk about it.

Prostate Cancer Physician, F, 54y

1.4 Socio-economic barriers to practise health behaviours

HP's perceived practical barriers for survivors included affordability of a healthy diet or gym subscription. Furthermore, perceived lack of social support and cultural variation were also reported as a barrier to practising health behaviours.

I think the difficulty is that depending on the demographics of patients you've got how practical it can be to either give the information or encourage people to follow the guidelines. As I say, our demographics are such that there's a lot of poverty within our catchment area. So that's often the biggest barrier to doing that. There's also… depending on cultural backgrounds, some of our patients are not keen to take responsibility, they prefer the paternalistic approach. So that can be quite difficult. We also have a lot of patients who are very socially isolated, and are not inclined to go shopping, or cook for themselves.

Colorectal Cancer Nurse, F, 55y

2. Clinician-centred barriers to provision of lifestyle advice

2.1 Knowledge and attitudes towards evidence and guidelines for health behaviours

HPs tended to be aware only of general lifestyle guidelines, such as the Department of Health alcohol guidelines, rather than specific guidelines for cancer survivors. Others were not aware of any guidelines.

I've come across research papers about things like that [smoking and diet], but I haven't come across any guidelines.

Physiotherapist, F, 51y

Their attitude towards the guidelines was positive but they based their advice only loosely on these. They described the guidelines for smoking and alcohol more accurately than those for physical activity or healthy eating.

For peer review only - http://bmjopen.bmj.com/site/about/guidelines.xhtml

> *I do take the guidance into consideration but I do a bit more patient-centred approach, really.*
>
> Colorectal Cancer Nurse, F, 51y

They also regarded the evidence on healthy lifestyle, cancer prevention, and cancer recurrence as not definitive. They perceived the evidence for physical activity to be stronger than that available for any dietary factors; however, they were still advocates of a healthy diet for overall health. There were mixed views regarding the role of lifestyle in cancer recurrence, with only some professionals aware of this link and research findings.

> *Well, I suppose that it helps to reduce the risk of recurrence. A good healthy diet and to take a good… you know, exercise.*
>
> Breast Cancer Physician, M, 40y

> *I wouldn't say that diet and physical activity are related to cancer recurrence.*
>
> Colorectal Cancer Physician, F, 41y

The fact that lifestyle is one of multiple cancer risk factors made them less inclined to focus their consultations on it.

> *There's obviously other factors at work and at play and, although I encourage patients to take exercise and to eat a healthy diet, particularly with lots of veg, I don't want to be creating a guilt feeling that, if they do all of this, it will stop it coming back. And therefore, if it comes back it's because they didn't eat the right diet. So I think it is important, but there's so many other factors involved that I don't want to concentrate on this to the detriment of others.*
>
> Colorectal Cancer Physician, F, 44y

In contrast, evidence to back claims about the impact of behaviour change was seen as both important to facilitate change and as something that patients do not pay attention to.

> *Perhaps it would be helpful for patients to have some information that specifically says: 'We know that patients who stop smoking… We know that patients who are more active… We know that patients who keep within a healthy weight…' and then it has those*

For peer review only - http://bmjopen.bmj.com/site/about/guidelines.xhtml

percentages for reduced risk of recurrence, etc. […] perhaps it would motivate them.

Breast Cancer Nurse, F, 64y

Clinicians also mentioned that they couldn't quantify the effect of lifestyle changes post-treatment, given the lack of research data. This made their argument weak and the conversation more challenging than those on medical treatments.

I can tell them if you take endocrine therapy, for survival, it would be improved by 10%. Chemotherapy, another 10%. So, 20% improvement. So there is some evidence for them for me to talk to the patient with the other treatments I'm offering, but I don't have anything for these [healthy lifestyle]. So that's another reason I won't be able to give them full information, that's when I say lack of information to give to the patients.

Breast Cancer Surgeon, M, 44y

2.2 Self-identification as the right person to provide lifestyle advice

While some felt it was their duty to empower survivors to lead a healthier lifestyle, others thought that they were not the right person to provide lifestyle advice, given their limited knowledge on the topic and other priorities during their short consultations.

And I think we have a duty to advise patients on quitting smoking and reducing alcohol intake and stuff.

Colorectal Cancer Nurse, F, 38y

I don't think it's necessarily my role to provide generic lifestyle advice to everybody, and unfortunately there isn't the time. I tend to focus on the people who have a problem, like fatigue, which I think would respond to exercise.

Prostate Cancer Physician, F, 34y

Some HPs thought their authority might help survivors take the advice more seriously, while others perceived their advice to be mostly ineffective.

I don't think that doctors necessarily are the best [to provide advice] unless they are felt to be a, sort of, place of authority for the patient.

Prostate Cancer Physician, F, 54y

For peer review only - http://bmjopen.bmj.com/site/about/guidelines.xhtml

There aren't many people I've advised to stop smoking who have ever done it. So I'm fairly sceptical about the value of my advice even though I persist with it.

Breast Cancer Physician, M, 64y

They perceived their own lack of adherence to lifestyle guidelines as both a barrier and a facilitator for providing advice; by not being a role model and by being able to relate to the challenge of behaviour change during the conversation with the survivors, respectively.

I don't have a BMI under 25, so I have no room to talk. Sometimes, though, a bit of 'I know how you feel' and a bit of feeling goes a long way. I know that one of my breast cancer nurses, who is very slim and very athletic, finds it far more difficult to talk to people about losing weight, because she doesn't feel she has anywhere to come from.

Breast Cancer Surgeon, F, 53y

2.3 Practical barriers to lifestyle advice provision

HPs regarded lack of time as a significant barrier for provision of lifestyle advice.

I don't have enough time to do it all properly and sensitively.

Colorectal Cancer Physician, F, 44y

They mentioned that they mostly provide verbal lifestyle advice due to a lack of other type of resources. Some tended to reiterate this advice in their patient letters, provide leaflets generated by their local multidisciplinary team, or refer people to existing relevant services for further support.

If some patients I think will benefit from a proper programme, then we give them this Living Well leaflet. If some patients who are saying they only have exercise and they are happy with it, then we don't given them that Living Well programme or healthy lifestyle leaflet. Apart from that, I don't have any other leaflets about diet or exercise as such, a specific how it improves their breast cancer survival. So in those questions I don't have nothing much to give them. And in my letter, like one line, I'll just mention to the patient the importance of maintaining a healthy weight, diet, and exercise.

Breast Cancer Surgeon, M, 44y

For peer review only - http://bmjopen.bmj.com/site/about/guidelines.xhtml

3. Optimal delivery of lifestyle advice

3.1 Tailored advice delivered throughout the cancer journey

Clinicians stressed that there is not a one-size-fit-all optimal format of lifestyle advice and were keen on providing choice of multiple formats, so that the patients would choose the one that fits best their individual needs and preferences. This was thought to enhance intervention adherence.

> *The level of intervention will depend on the patients, their background, the sort of feedback they give us to their motivation.*
>
> Prostate Cancer Physician, M, 49y

> *I think the best format is that somebody talks to the patient and asks them what they would want, and then plan the intervention accordingly rather than having one fitting for everybody, because that's not going to help.*
>
> Prostate Cancer Physician, F, 54y

The views on the optimal timing of providing advice were varied with some suggesting to intervene at diagnosis and others at the end of treatment. Some suggested that the optimal timing is patient-dependent. Others felt that the advice should be initiated at diagnosis and reiterated in each follow-up appointment.

> *It's something that should have been on the agenda all the way through and then [at the end of treatment] you're literally summarising, concluding, and then exploring if there's something they want to take over to another level..*
>
> Prostate Cancer Physician, M, 49y

Clinicians mentioned that a combination of verbal and written lifestyle information would be preferable to only verbal advice, as patients receive too much information during their cancer journey and they cannot take in everything. This was viewed as preferable to written advice alone as it could be made more relevant to the patient. They felt that all HPs should provide lifestyle advice.

> *If I just gave them a leaflet… I think it needs to be given in conjunction with a health professional or somebody who can talk them through it. If you just give them another leaflet, they're just likely to put it with their pack of other things they've got.*
>
> Physiotherapist, F, 51y

For peer review only

For peer review only - http://bmjopen.bmj.com/site/about/guidelines.xhtml

Those in favour of advice using a face-to-face meeting with a HP felt that this meeting should be part of the general follow-up to boost attendance and acceptability. They mentioned that the meeting could enhance continuity of care post-treatment, fostering personal contact with a professional aware of the patient's journey. For the survivors who would prefer the support of a group-based intervention, local community venues were preferred over hospital rooms. Online advice was deemed appropriate for technology-literate survivors.

> *A group intervention is definitely a positive thing for patients to get that peer support from other patients as well. I think it helps patients to feel that they can manage their condition better themselves and also with support from other patients rather than it just being medically led all the time, particularly if it's off site as well, if it's in other, perhaps, community settings as well.*
>
> Breast Cancer Nurse, F, 46y

> *I think online is probably the best thing, if patients have access and they're computer literate, because they can do it at their own time and at their own pace.*
>
> Colorectal Cancer Nurse, F, 55y

3.2 Small and achievable changes framed as part of their treatment regimen

HPs mentioned that survivors who need to change multiple behaviours might be overwhelmed by the process and suggested prioritising and tackling one or two behaviours at a time focusing on small realistic changes.

> *I'm aware that if you give people too much advice all at once, it's quite difficult for them to take it all in, so sometimes you are better off just focusing on one thing […] finding out which you think is the most risky of their behaviours and concentrate on that one.*
>
> Prostate Cancer Nurse, F, 53y

Some thought that framing the healthy lifestyle advice as a treatment regimen would make it more engaging.

> *It would be nice to have some guidelines available, some specific booklet available about benefits of healthy diet and exercise, so we can give it to them as well, so they can follow it as a specific*

For peer review only

treatment regimen. [...] It is a treatment – healthy diet and exercise – and say it's a treatment. We should make them feel like that.

Breast Cancer Surgeon, M, 44y

3.3 Cost-effectiveness for wide-scale implementation

Time and financial resources and proven cost-effectiveness were cited as barriers for wide-scale implementation of lifestyle advice. Lower need for resources indicated higher potential for implementation but clinicians were unsure of measurable outcomes for one-off low-intensity lifestyle interventions, such as a leaflet or a phone call.

I think the patients would like the face-to-face. I suppose it just depends on what the resources are, as to who is doing it – is it a dietitian, is it a CNS, is it another organisation? So I think it depends on the manpower, what resources are available for a face-to-face. I think if it was extra on top of what we currently provide then we would struggle.

Colorectal Cancer Nurse, F, 54y

Integration is fine [for leaflet and online advice]. The question is: will it help?

Prostate Cancer Physician, F, 54y

Discussion

The current study reiterated that HPs' desire to provide lifestyle advice was not necessarily matched by knowledge and action. Barriers to provision included perceptions about survivor's behaviours, abilities and interest in advice, as well as concerns about HP's own abilities to provide effective advice without damaging their patient relationships. Nonetheless, HPs were keen for most survivors to receive lifestyle advice throughout the cancer journey, tailored to their individual needs and preferences, by initiating small and achievable change goals framed as part of their treatment regimen.

Previously reported barriers to provision of lifestyle advice include perception that patients lack interest in behaviour change, perception that behaviour change is not feasible or effective, lack of guidelines and confusion about the research findings, self-identification as not the right person to provide advice, potential for blaming the

For peer review only - http://bmjopen.bmj.com/site/about/guidelines.xhtml

For peer review only

patient, lack of appropriate behaviour change support, and lack of time.[16 19 20] The current study confirmed the above barriers offering further insight, such as the perception that providing advice on alcohol and smoking is more challenging compared to diet and physical activity. Another insight was that the fear of blaming relates not only to cancer onset but also to cancer recurrence. The study also added to the list of barriers the potential loss of credibility and connection with survivors, the obstruction of free will, the importance of role modelling, and the challenge of addressing the co-existence of multiple suboptimal behaviours given practical barriers to implementing advice, such as affordability of a healthy diet. These factors should be taken into consideration in the development of programmes aiming to train HPs to effectively deliver lifestyle advice, as evidence suggests that even brief advice from HPs is effective for lifestyle behaviour change.[21-23]

Interestingly, the HPs' self-identification as not the right person to provide advice and their fear of losing the connection with their patients by providing them with continuous lifestyle advice, are not matched by the fact that survivors would actually welcome lifestyle advice from their oncology HPs.[13-15] Survivors' views partly support the perception of HPs that it is difficult to change engrained unhealthy lifelong habits. However, although there is little evidence that survivors make major changes in their health behaviours following a cancer diagnosis,[12] some report motivation to engage in behaviour change.[13 24] HP's therefore have an opportunity to capitalise on this and to provide lifestyle advice to survivors or signpost them to further support. These interventions should include social support, because this behaviour change technique was perceived to facilitate performing health behaviours by both HPs in the current study and survivors.[15]

However, HPs in this study did not mention reputable and long-standing lifestyle guidelines for cancer survivors.[11] One reason for the lack of awareness might be that these guidelines are based on the cancer prevention literature. However, the evidence base for their implementation post-diagnosis is growing, and they are considered to be prudent advice for cancer survivors. The lack of dissemination of these guidelines should therefore be targeted with educational courses, conferences, and, training to enable HPs to provide evidence-based advice. Professional organisations are likely to be pivotal in this process. Programmes to enhance

For peer review only

oncology HPs knowledge on lifestyle guidelines and facilitate provision of advice are currently being piloted, and are showing promising potential.[25 26] These programmes should ensure not only adequate knowledge of the guidelines but also provide the tools and techniques to HPs to ensure they approach the topic of behaviour change in a supportive way with confidence and sensitivity, so that cancer survivors feel motivated and not guilty.

HPs in this study also indicated that the format of lifestyle advice should be tailored to individual survivors. Surveys, qualitative studies, and analysis of data from large behaviour change interventions are required to identify factors that predict cancer survivors' uptake and adherence to various types of lifestyle interventions. However, an analysis of three large home-based lifestyle interventions in cancer survivors did not identify any predictors of adherence to the different types of intervention.[27] To that end, quasi-experimental intervention designs (e.g. face-to-face vs. remote intervention) based on survivors' preferences for delivery format might better facilitate understanding of adherence and lead to effective personalised lifestyle support. Framing the intervention as part of the treatment regimen instead of health promotion advice should also be explored in future studies, with pilot studies indicating the effectiveness of physical activity rehabilitation on fitness levels.[28]

Strengths of the current study include the comprehensive examination of HPs' views on all major lifestyle topics from a sample of various HPs from across the UK. Although qualitative studies provide in-depth information, their findings cannot be generalised. It is possible that the participants of this study were generally interested in lifestyle topics, which might bias the responses towards a positive view on lifestyle. Furthermore, the views may not be representative of all oncology HPs, as we aimed to target those working on the most common lifestyle-related cancers. Further research can explore this, as it might be even less likely for HPs working with other cancers to give advice.

In conclusion, the findings of this study suggest that HPs specialising in breast, prostate and colorectal cancers, tend to provide generic advice broadly based on lifestyle guidelines and try to tailor it to the individual patient. Additional education is needed to improve awareness of available guidelines for cancer survivors.

For peer review only

For peer review only - http://bmjopen.bmj.com/site/about/guidelines.xhtml

Incorporation of the identified factors that influence the provision of lifestyle advice in the development of training programmes for HPs and lifestyle interventions for cancer survivors could increase the probability of effectiveness and in turn, improve outcomes for cancer survivors.

For peer review only

For peer review only - http://bmjopen.bmj.com/site/about/guidelines.xhtml

Acknowledgements

All authors were supported by funding from Cancer Research UK. SL was also funded by University College London and the Weight Concern Impact Award Studentship. RJB is currently supported by Yorkshire Cancer Research Academic Fellowship funding. The researchers are grateful to the health professionals that participated in the study.

Declaration of Conflicting Interests

The authors declare no conflict of interests.

Contributorship statement

RJB conceived the study and obtained the funding for the Impact Award Studentship. SL, RJB, AF, KW, and HC devised the interview protocol. SL conducted the interviews. SL, DAK, and RJB analysed the data with contribution from all authors. DAK drafted the manuscript. All authors critically revised, and approved the final version of the manuscript.

Data sharing

Anonymised interview transcripts can be obtained by the corresponding author on reasonable request.

For peer review only

For peer review only - http://bmjopen.bmj.com/site/about/guidelines.xhtml

References

1. Maddams J, Utley M, Moller H. Projections of cancer prevalence in the United Kingdom, 2010-2040. Br J Cancer 2012;**107**(7):1195-202.
2. Quaresma M, Coleman MP, Rachet B. 40-year trends in an index of survival for all cancers combined and survival adjusted for age and sex for each cancer in England and Wales, 1971-2011: a population-based study. Lancet 2015;**385**(9974):1206-18.
3. Keats MR, Cui Y, Grandy SA, et al. Cardiovascular disease and physical activity in adult cancer survivors: a nested, retrospective study from the Atlantic PATH cohort. J Cancer Surviv 2017;**11**(2):264-73.
4. Duan W, Li S, Meng X, et al. Smoking and survival of breast cancer patients: A meta-analysis of cohort studies. Breast 2017;**33**:117-24.
5. Schwedhelm C, Boeing H, Hoffmann G, et al. Effect of diet on mortality and cancer recurrence among cancer survivors: a systematic review and meta-analysis of cohort studies. Nutr Rev 2016;**74**(12):737-48.
6. Stacey FG, James EL, Chapman K, et al. A systematic review and meta-analysis of social cognitive theory-based physical activity and/or nutrition behavior change interventions for cancer survivors. J Cancer Surviv 2015;**9**(2):305-38.
7. Stolley M, Sheean P, Gerber B, et al. Efficacy of a Weight Loss Intervention for African American Breast Cancer Survivors. J Clin Oncol 2017:JCO2016719856.
8. Rock CL, Doyle C, Demark-Wahnefried W, et al. Nutrition and physical activity guidelines for cancer survivors. CA Cancer J Clin 2012;**62**(4):243-74.
9. Schmitz KH, Courneya KS, Matthews C, et al. American College of Sports Medicine roundtable on exercise guidelines for cancer survivors. Med Sci Sports Exerc 2010;**42**(7):1409-26.
10. WCRF. Continuous Update Project Report: Diet, Nutrition, Physical Activity, and Breast Cancer Survivors: World Cancer Research Fund International, 2014.
11. WCRF. Food, Nutrition, Physical Activity and the Prevention of Cancer: A Global Perspective. Washington, DC: World Cancer Research Fund / American Institute for Cancer Research, 2007.
12. Williams K, Steptoe A, Wardle J. Is a cancer diagnosis a trigger for health behaviour change? Findings from a prospective, population-based study. Br J Cancer 2013;**108**(11):2407-12.
13. Beeken RJ, Williams K, Wardle J, et al. "What about diet?" A qualitative study of cancer survivors' views on diet and cancer and their sources of information. Eur J Cancer Care (Engl) 2016;**25**(5):774-83.
14. Koutoukidis DA, Beeken RJ, Lopes S, et al. Attitudes, challenges, and needs about diet and physical activity in endometrial cancer survivors: a qualitative study. Eur J Cancer Care (Engl) 2016.
15. Smith L, Croker H, Fisher A, et al. Cancer survivors' attitudes towards and knowledge of physical activity, sources of information, and barriers and facilitators of engagement: A qualitative study. Eur J Cancer Care (Engl) 2017;**26**(4).
16. Williams K, Beeken RJ, Fisher A, et al. Health professionals' provision of lifestyle advice in the oncology context in the United Kingdom. Eur J Cancer Care (Engl) 2015;**24**(4):522-30.
17. Bengtsson M. How to plan and perform a qualitative study using content analysis. NursingPlus Open 2016;**2**(Supplement C):8-14.

For peer review only - http://bmjopen.bmj.com/site/about/guidelines.xhtml

18. Tong A, Sainsbury P, Craig J. Consolidated criteria for reporting qualitative research (COREQ): a 32-item checklist for interviews and focus groups. Int J Qual Health Care 2007;**19**(6):349-57.
19. Miles A, Simon A, Wardle J. Answering patient questions about the role lifestyle factors play in cancer onset and recurrence: what do health care professionals say? Journal of health psychology 2010;**15**(2):291-8.
20. Sutton E, Hackshaw-McGeagh LE, Aning J, et al. The provision of dietary and physical activity advice for men diagnosed with prostate cancer: a qualitative study of the experiences and views of health care professionals, patients and partners. Cancer causes & control : CCC 2017;**28**(4):319-29.
21. NICE. Physical activity: brief advice for adults in primary care. In: Excellence NIfHaC, ed., 2013.
22. Jackson SE, Wardle J, Johnson F, et al. The impact of a health professional recommendation on weight loss attempts in overweight and obese British adults: a cross-sectional analysis. BMJ Open 2013;**3**(11):e003693.
23. Fisher A, Williams K, Beeken R, et al. Recall of physical activity advice was associated with higher levels of physical activity in colorectal cancer patients. BMJ Open 2015;**5**(4):e006853.
24. Anderson AS, Steele R, Coyle J. Lifestyle issues for colorectal cancer survivors--perceived needs, beliefs and opportunities. Supportive care in cancer : official journal of the Multinational Association of Supportive Care in Cancer 2013;**21**(1):35-42.
25. Webb J, Hall J, Hall K, et al. Increasing the frequency of physical activity very brief advice by nurses to cancer patients. A mixed methods feasibility study of a training intervention. Public health 2016;**139**:121-33.
26. Murphy J, Worswick L, Pulman A, et al. Translating research into practice: evaluation of an e-learning resource for health care professionals to provide nutrition advice and support for cancer survivors. Nurse education today 2015;**35**(1):271-6.
27. Adams RN, Mosher CE, Blair CK, et al. Cancer survivors' uptake and adherence in diet and exercise intervention trials: an integrative data analysis. Cancer 2015;**121**(1):77-83.
28. West MA, Loughney L, Lythgoe D, et al. Effect of prehabilitation on objectively measured physical fitness after neoadjuvant treatment in preoperative rectal cancer patients: a blinded interventional pilot study. British journal of anaesthesia 2015;**114**(2):244-51.

For peer review only

For peer review only - http://bmjopen.bmj.com/site/about/guidelines.xhtml

Supplementary material 1: Interview Topic Guide

	Topic	Question	Prompt
1.	Introductions / background	Brief introductions; get verbal consent to tape-record the interview; remind the aims of study, check length of interview/audio-recorded.	
2.	Health beliefs	What are your views on the role of diet and physical activity in the recovery from [breast/prostate/colorectal] cancer?<br>What are your views on the role of diet and physical activity in cancer recurrence?	
3.	Awareness of lifestyle guidelines for cancer survivors	Are you familiar with any guidelines specifically for cancer patients for any of the following lifestyle topics?<br>You said that you were aware of “X, Y, Z” guidelines. Can you please tell me what you know (or what you remember) about each of these guidelines?	Prompt<br>- PA<br>- Diet<br>- Weight management<br>- Smoking<br>- Alcohol
		What are your views on these guidelines? What do you think about them?	Prompt<br>- Liking<br>- Realistic<br>- Reliable
		How did you find out about them?<br>You also said that you were not aware of the “K, M” guidelines. Why do you think that you did not come across these guidelines?<br>What were the barriers for you not getting this information?	
3.	Barriers for provision of lifestyle advice	Thinking of all your patients who have completed primary treatment for cancer, do you give your patients advice on any lifestyle topics?	Prompt<br>- PA<br>- Diet<br>- Weight management<br>- Smoking<br>- Alcohol
		You said that you give advice on “X lifestyle topic” to “X%” of your patients. What kind of advice do you tend to give?	Prompt<br>- Verbal

For peer review only

Topic	Question	Prompt
		- Written<br>- Website referral<br>- HP referral<br>- When / Why this specific format<br>- Guidelines based
	Why do you think that you provide advice on this to [so many / only to x%] of your patients?<br>What prevents you from providing this type of advice to more patients?<br>What are the reasons why you don't usually provide lifestyle advice?	Prompt<br>- Verbal<br>- Written<br>- Website referral<br>- HP referral
	For each lifestyle topic that professional does NOT provide any advice, ask: Is there any particular reason why you have not given advice on "X lifestyle topic" to any of your patients?<br>What are the barriers preventing you to provide this advice?	Prompt<br>- Guidelines
	On the survey, we asked you about a number of specific barriers preventing you to provide lifestyle advice for your patients. You mentioned that "X" and "Y" were the main barriers for you. Can you tell me more about these barriers? Why these prevent you from providing lifestyle advice?<br>Are there any other barriers?	
5. Content Expectations	Have any of your cancer patients ask you information about lifestyle?<br><br>What type of information do they usually ask for?<br>How often do they ask you about lifestyle information? Why?<br>Is there any other type of information they should be getting? Why do you think it is important they get that information?<br>Is there anything you would avoid in telling them? Why?	

For peer review only

Topic	Question	Prompt
	Have any of the relatives, family members, friends or carers of your patients asked you information about lifestyle?<br>What type of information do they usually ask for?<br>How often do they ask you about lifestyle information? Why?<br>Is there any other type of information they should be getting? Why do you think it is important they get that information?<br>Is there anything you would avoid in telling relatives, friends or carers? Why?	
6. Delivery	We are planning to develop some materials to provide information on diet and physical activity for people who have had cancer treatment.<br>When would it be the best time for health professionals to provide information on healthy eating and physical activity to colorectal cancer patients? Why?<br>Do you think patients would be open to receive this type of information at that time? Why?<br>Who should provide them information about healthy eating and physical activity? Why?<br>In what format do you think that it would be better to provide information about diet and physical activity, for colorectal cancer patients? Why?	
	What do you think about a leaflet?<br>What do you think about a leaflet along with a telephone call from a HP?<br>What do you think about a leaflet along with a face-to-face contact with a HP?<br>What do you think about a leaflet along with online information?<br>What do you think about a self-help book with specific information for cancer survivors?<br>What do you think about a group intervention designed for cancer survivors with about 8 sessions, providing more detailed lifestyle information?<br>We have discussed about several options and formats to provide lifestyle information. Balancing between what would be better for HPs, for patients and feasibility of implementation; what do you think would be the best format to provide lifestyle advice?	Prompt<br>- Usefulness<br>- Receipt<br>- Implementation
	From your perspective, what are the issues or barriers to take into account that some [breast/prostate/colorectal] cancer patients might face in trying to change their	Prompt<br>- Mood

Topic	Question	Prompt
	lifestyle?	- Mental health<br>- Physical restrictions<br>- Visual problems
7. Ending	Do you have anything else to add, any comments or any questions?	

For peer review only

Supplementary material 2 – Interviews NVIVO Coding Framework

(1) Health Beliefs

- Beliefs about role of diet in cancer (incl. cancer recurrence)
- Beliefs about role of exercise in cancer (incl. cancer recurrence)
- Beliefs about role of alcohol in cancer (incl. cancer recurrence)
- Beliefs about role of smoking in cancer (incl. cancer recurrence)
- Health beliefs (not lifestyle)

(2) Awareness of lifestyle guidelines for cancer survivors

- Aware of guidelines (& description)
- Not aware of guidelines (& description)
- Views on guidelines
- Barriers to accessing guidelines (incl. not aware)
- How found out about guidelines

(3) Provision of lifestyle advice

- Advice given (diet)
- Advice given (exercise)
- Advice given (smoking)
- Advice given (alcohol)
- Advice given (weight)
- Advice given and requested by Relatives
- Advice would like to get on other topics
- Barriers to provision of advice
- Reason for provision of advice to a HIGH number of pts
- Reason for provision of advice to a LOW number of pts

(4) Format

- Timing
  - Timing HP has provided lifestyle advice
  - Preferred timing for provision of lifestyle advice
- Source

For peer review only

Mode of delivery (all negative, incl. leaflet, self-help book and group)

Ideal Mode of delivery

Mode of delivery of information provided (incl verbally, referral, web, leaflet, self help and group)

Format Group

Format Verbally

Format Digital

Format Written (All)

Leaflet only

Leaflet + telephone call

Leaflet + face-to-face contact

Leaflet + online information

Self-Help book

(5) Barriers to attending lifestyle interventions - internal & external

(6) Pathway issues

Leaflet only

Leaflet + telephone call

Leaflet + face to face contact

Leaflet + online information

Self-Help Book

Group intervention

Other Pathway factors

For peer review only

COREQ (COnsolidated criteria for REporting Qualitative research) Checklist

A checklist of items that should be included in reports of qualitative research. You must report the page number in your manuscript where you consider each of the items listed in this checklist. If you have not included this information, either revise your manuscript accordingly before submitting or note N/A.

Topic	Item No.	Guide Questions/Description	Reported on Page No.
**Domain 1: Research team and reflexivity**			
Personal characteristics			
Interviewer/facilitator	1	Which author/s conducted the interview or focus group?	5
Credentials	2	What were the researcher's credentials? E.g. PhD, MD	5
Occupation	3	What was their occupation at the time of the study?	5
Gender	4	Was the researcher male or female?	5
Experience and training	5	What experience or training did the researcher have?	5
Relationship with participants			
Relationship established	6	Was a relationship established prior to study commencement?	5
Participant knowledge of the interviewer	7	What did the participants know about the researcher? e.g. personal goals, reasons for doing the research	5
Interviewer characteristics	8	What characteristics were reported about the inter viewer/facilitator? e.g. Bias, assumptions, reasons and interests in the research topic	5
**Domain 2: Study design**			
Theoretical framework			
Methodological orientation and Theory	9	What methodological orientation was stated to underpin the study? e.g. grounded theory, discourse analysis, ethnography, phenomenology, content analysis	5
Participant selection			
Sampling	10	How were participants selected? e.g. purposive, convenience, consecutive, snowball	5
Method of approach	11	How were participants approached? e.g. face-to-face, telephone, mail, email	5
Sample size	12	How many participants were in the study?	6
Non-participation	13	How many people refused to participate or dropped out? Reasons?	6
Setting			
Setting of data collection	14	Where was the data collected? e.g. home, clinic, workplace	5
Presence of non-participants	15	Was anyone else present besides the participants and researchers?	N/A
Description of sample	16	What are the important characteristics of the sample? e.g. demographic data, date	6
Data collection			
Interview guide	17	Were questions, prompts, guides provided by the authors? Was it pilot tested?	5
Repeat interviews	18	Were repeat inter views carried out? If yes, how many?	N/A
Audio/visual recording	19	Did the research use audio or visual recording to collect the data?	5
Field notes	20	Were field notes made during and/or after the inter view or focus group?	5
Duration	21	What was the duration of the inter views or focus group?	5
Data saturation	22	Was data saturation discussed?	N/A
Transcripts returned	23	Were transcripts returned to participants for comment and/or	N/A

For peer review only

Topic	Item No.	Guide Questions/Description	Reported on Page No.
		correction?	
**Domain 3: analysis and findings**			
Data analysis			
Number of data coders	24	How many data coders coded the data?	6
Description of the coding tree	25	Did authors provide a description of the coding tree?	Appendix 2
Derivation of themes	26	Were themes identified in advance or derived from the data?	5-6
Software	27	What software, if applicable, was used to manage the data?	5
Participant checking	28	Did participants provide feedback on the findings?	N/A
Reporting			
Quotations presented	29	Were participant quotations presented to illustrate the themes/findings? Was each quotation identified? e.g. participant number	6-15
Data and findings consistent	30	Was there consistency between the data presented and the findings?	6-15
Clarity of major themes	31	Were major themes clearly presented in the findings?	6-15
Clarity of minor themes	32	Is there a description of diverse cases or discussion of minor themes?	6-15

Developed from: Tong A, Sainsbury P, Craig J. Consolidated criteria for reporting qualitative research (COREQ): a 32-item checklist for interviews and focus groups. *International Journal for Quality in Health Care*. 2007. Volume 19, Number 6: pp. 349 – 357

Once you have completed this checklist, please save a copy and upload it as part of your submission. DO NOT include this checklist as part of the main manuscript document. It must be uploaded as a separate file.

For peer review only

BMJ Open

Lifestyle advice to cancer survivors: a qualitative study on the perspectives of health professionals

Journal:	*BMJ Open*
Manuscript ID	bmjopen-2017-020313.R1
Article Type:	Research
Date Submitted by the Author:	12-Jan-2018

Complete List of Authors:	Koutoukidis, Dimitrios; University College London, Behavioural Science and Health; University of Oxford, Nuffield Department of Primary Care Health Sciences<br>Lopes, Sonia; University College London, Behavioural Science and Health<br>Fisher, Abigail; University College London, Behavioural Science and Health<br>Williams, Kate; University College London, Behavioural Science and Health<br>Croker, Helen; University College London, Behavioural Science and Health<br>Beeken, Rebecca; University College London, Behavioural Science and Health; University of Leeds Leeds Institute of Health Sciences

**Primary Subject Heading**:	Oncology
Secondary Subject Heading:	Qualitative research
Keywords:	health professionals, cancer survivors, lifestyle, guidelines, interventions

SCHOLARONE™ Manuscripts

Lifestyle advice to cancer survivors: a qualitative study on the perspectives of health professionals

Dimitrios A. Koutoukidis[1,2¶], Sonia Lopes[1¶], Abigail Fisher[1], Kate Williams[1], Helen Croker[1], Rebecca J. Beeken[1,3]

[1]Department of Behavioural Science and Health, University College London, London, UK

[2]Nuffield Department of Primary Care Health Sciences, University of Oxford, Oxford, UK

[3]Leeds Institute of Health Sciences, University of Leeds, Leeds, UK

[¶]Contributed equally

Corresponding Author:

Dr Rebecca Beeken

Leeds Institute of Health Sciences

Level 10, Worsley Building,

Clarendon Way,

Leeds, LS2 9NL Tel. +44 (0)113 343 3228 E-mail: r.beeken@leeds.ac.uk

For peer review only

For peer review only - http://bmjopen.bmj.com/site/about/guidelines.xhtml

Abstract

Objectives: Adoption of healthy lifestyle behaviours has shown promising effectiveness in reducing the high morbidity burden of cancer survivors. Health professionals (HPs) are well suited to provide lifestyle advice but few survivors report receiving guidance from them. This study aimed to explore HPs' perspective of lifestyle advice (on heathy eating, physical activity, smoking, and alcohol) for cancer survivors.

Design: In-depth semi-structured qualitative interviews were conducted by telephone or face-to-face. Data were analysed using qualitative content analysis.

Setting and participants: Twenty-one UK HPs working in secondary care with breast, prostate, or colorectal cancer survivors were interviewed.

Results: The over-arching theme was that HPs' desire to provide lifestyle advice was not necessarily matched by knowledge and action. Three main themes were identified: 1) survivorship-centred barriers to provision, 2) HP-centred barriers to provision, and 3) optimal delivery of lifestyle advice. Results suggested that HPs' perceptions of survivors' current status of practising health behaviours, their perceived socio-economic barriers and ability to practise health behaviours, and HPs' fear for potential loss of connection with the patient influenced provision of lifestyle advice. Further factors included HPs' knowledge of healthy lifestyle guidelines, feeling that they were not the 'right person' to provide advice, and lack of time and resources. HPs stressed that the optimal delivery of lifestyle advice should (a) be tailored to the individual and delivered throughout the cancer journey, (b) be focused on small and achievable changes framed as part of their treatment regimen, and (c) be cost-effective for wide-scale implementation.

Conclusions: Incorporation of the identified barriers when developing HP training programmes and lifestyle interventions could increase the probability of successful behaviour change and, thus, improved outcomes for cancer survivors.

Keywords: health professionals, cancer survivors, lifestyle, guidelines, interventions

For peer review only - http://bmjopen.bmj.com/site/about/guidelines.xhtml

Strengths and limitations of the study

- The study comprehensively examined the views of health professionals, working with breast, prostate, and colorectal cancer survivors, on key modifiable lifestyle behaviours.
- Health professionals had diverse professional backgrounds, including nursing, oncology, surgery, and allied health professions.
- It is possible that the participants were generally interested in lifestyle topics, which might bias the responses towards a positive view on lifestyle.
- Although qualitative studies provide in-depth information, their findings cannot be generalised.

For peer review only

For peer review only - http://bmjopen.bmj.com/site/about/guidelines.xhtml

Introduction

There are 2.5 million cancer survivors living in the UK.[1] The increasing number of cancer survivors and their higher morbidity burden compared to the general population means strategies are needed to improve survivorship outcomes.[2 3] Adoption of healthy behaviours, such as not smoking or drinking alcohol, eating a healthy diet, engaging in sufficient physical activity, and weight management, are potential strategies. There is a growing body of evidence that suggests these health behaviours are positively associated with improved outcomes in cancer survivors.[4 5] Furthermore, behavioural lifestyle interventions in this population have shown promising effectiveness.[6 7]

There are now lifestyle guidelines for cancer survivors, advocating smoking cessation, moderate alcohol consumption (if any), a healthy balanced diet, daily physical activity, and maintenance of a healthy weight.[8-11] Although surveys suggest that few cancer survivors are meeting these guidelines,[12] qualitative data suggest that a cancer diagnosis may promote motivation for behaviour change and that survivors are keen to receive healthy lifestyle advice, ideally from a credible source.[13 14] Health professionals (HPs) are well positioned to provide healthy lifestyle advice and, thus, help cancer survivors improve their lifestyle behaviours. However, few survivors report receiving lifestyle advice from their HPs.[13 15]

We previously conducted a survey with HPs suggesting that 36% of HPs were not aware of any lifestyle guidelines for cancer survivors.[16] Although most reported providing some lifestyle advice, a number of barriers to provision were also cited, including lack of guidelines, belief that advice would not change behaviour or affect outcomes, belief that they are not the right person to provide advice, lack of time, lack of patient interest, fear that they would offend the patient, and cultural differences. These highlighted the potential for an intervention to help HPs provide evidence-based advice.

The aim of this qualitative study was to explore in greater depth HPs' views on the provision of lifestyle advice to patients with a cancer diagnosis.

Methods

For peer review only - http://bmjopen.bmj.com/site/about/guidelines.xhtml

Procedure and Participants

Eligible participants were UK-based HPs (including surgeons, physicians, nurses and allied HPs) caring for patients diagnosed with the most common lifestyle-related cancers (breast, prostate, or colorectal cancer). They had provided contact details following their participation in an online survey on lifestyle guidelines for cancer survivors and agreed to be contacted for future research.[16] HPs were invited via e-mail to participate in either a telephone (n=20) or an individual face-to-face (n=1) interview at University College London. Participants provided signed informed consent. Ethical approval was granted by the UCL Research Ethics Committee (project ID: 4456/001).

Interviews

Between October 2014 and January 2015, an experienced female research psychologist with interest in cancer survivorship (SL, MSc) interviewed the HPs. The interviewer had no previous relationship with the study participants. Participants were aware that the study was part of the interviewer's research degree. Each interview lasted approximately 40 minutes (range: 17-74 minutes). The interview followed a semi-structured guide (Supplementary material 1). The guide followed up on HPs' individual survey responses about the provision of advice to patients who had completed treatment, and used open-ended questions and prompts to explore in-depth HPs' awareness of survivorship lifestyle guidelines, their barriers to provision of lifestyle advice, and their views on various formats for lifestyle interventions. Interviews were audio-recorded, anonymised, and transcribed verbatim. The researcher listened to the tapes to verify them for accuracy. Field notes were not taken as all but one interview took place over the phone, meaning information could not be collected on setting, appearance or nonverbal behaviours of participants.

Analysis

Interview transcripts were analysed using qualitative content analysis[17] in NVivo version 10. Content analysis was chosen as the main aim of the study was to describe HPs views as opposed to developing theory or exploring the lived experience[18]. Following familiarisation with the data, meaning units and codes were generated using an inductive approach. SL coded the transcripts derived the coding list (Supplementary material 2) in an iterative process with RJB. An independent

For peer review only - http://bmjopen.bmj.com/site/about/guidelines.xhtml

reviewer with a dietetics background (DAK) coded 20% of interviews against the coding list. Inter-rater reliability was high (mean kappa=0.92) and minor coding differences were resolved by discussion. Categories and themes were compiled with a manifest analysis using an iterative process. This ensured internal homogeneity and external heterogeneity of the themes and categories. The completed COREQ checklist on standard reporting guidelines is available in Supplementary material 3.[19]

Results

Sample Characteristics

Fifty-two of the 336 survey participants were eligible for this study. Twenty-one (40.4%) were interviewed and 31 either did not respond or were unwilling to take part. Table 1 shows the sample characteristics of the study in relation to the survey. Most participants were female and aged between 46 and 55 years. Their professional role varied and their work location spanned across the UK.

Table 1: Sample characteristics in relation to the previous survey

	Interview participants n=21	Survey participants n=336[1]
	% (n)	% (n)
Gender		
Female	76 (16)	81 (272)
Male	24 (5)	19 (64)
Age *(in years)*		
26-35	5 (1)	12 (41)
36-45	38 (8)	33 (110)
46-55	48 (10)	42 (141)
56-65	10 (2)	13 (42)
Profession		
Nurse	43 (9)	55 (126)
Physician	33 (7)	21 (48)
Surgeon	19 (4)	13 (31)
Allied HP (physiotherapist)	5 (1)	11 (26)
Cancer Specialism		
Breast	43 (9)	25 (54)
Colorectal	38 (8)	15 (32)
Prostate	19 (4)	10 (21)
Region of Work		
North West England	38 (8)	-

For peer review only

For peer review only - http://bmjopen.bmj.com/site/about/guidelines.xhtml

London	24 (5)	-
South West England	10 (2)	-
South East England	10 (2)	-
Wales	10 (2)	-
East of England	5 (1)	-
Scotland	5 (1)	-

[1] Data from Williams et al 2015. % may not total 100% due to rounding.

Themes

The overarching theme arising from the interviews was that HPs' desire to provide lifestyle advice was not necessarily matched by knowledge and action. Three main themes were identified: 1) HP's perceptions of survivor-centred barriers to provision of lifestyle advice, 2) HP-centred barriers to provision of lifestyle advice, and 3) optimal delivery of lifestyle advice.

1 HPs' perceptions of survivor-centred barriers to provision of lifestyle advice

1.1. Perceptions of survivors' current health behaviours

HPs felt that lifestyle behaviour change was particularly challenging for cancer survivors, due to ingrained lifelong habits and sustainability of achieved changes.

> *Well, lifestyle is very much a matter of habit, and breaking habits is always difficult for all of us.*
>
> Breast Cancer Surgeon, M, 63y

Co-existence of multiple sub-optimal behaviours was regarded as a barrier to provision of lifestyle advice.

> *It is sometimes difficult when people just do seem to have all the vices, so they are heavy smokers, they are not just overweight, they are really obese, they obviously take no exercise and they are heavy smokers. It's very difficult to know sometimes where to start.*
>
> Breast Cancer Surgeon, F, 53y

Alcohol and tobacco were regarded as sensitive topics and it was perceived hard to assess consumption levels. Therefore, this was only addressed if there were evident indications of a problem or if the topic was raised by relatives.

> *[…] when it comes to alcohol it can be quite tricky. Sometimes we'll have relatives who will say, 'Are you aware this person is*

For peer review only

drinking?' particularly if they are on chemotherapy. And the side effects of chemotherapy, and then to add alcohol onto it. I think that's a more difficult area to try and challenge people on.

Colorectal Cancer Nurse, F, 54y

They felt that low mood and distress were also barriers for implementation of the provided advice.

If someone is depressed or has low mood, for whatever reason, everything is harder.

Breast Cancer Nurse, F, 40y

1.2 HPs' perception of survivors' ability to perform health behaviours

The lifestyle advice provided was mostly tailored to the individual patient. Frailty and physical inability to perform physical activity significantly hindered the HPs' inclination to provide physical activity advice. Tolerability of a high-fibre diet due to treatment-related bowel changes was also considered a barrier for provision of advice on healthy eating.

If somebody is frail and unwell generally, either because of their cancer or because of other medical problems, then I think advising them about diet and exercise and things isn't necessarily helpful for them.

Prostate Cancer Physician, F, 34y

I think some of our patients have got possibly some degree of stenosis, and actually saying to them to have a lot of fruit and fibre in their diet is wrong information. So, I think it has to be tailored to the individual, really.

Colorectal Cancer Nurse, F, 54y

1.3 Potential loss of connection with the patient

Fear of blaming the patient resulted in HPs taking a subtle rather than a strong approach to providing lifestyle advice. HPs did not want to make people feel guilty about their previous lifestyle or even create future blame if the patient did not subsequently change their lifestyle and the cancer reoccurred. They mentioned a potential loss of credibility and connection with their patients if they were to insist on lifestyle change.

For peer review only - http://bmjopen.bmj.com/site/about/guidelines.xhtml

I need to keep credibility with them as well, and if I start making demands of them which they really can't possibly keep then I kind of lose my own connection with these people.

Breast Cancer Surgeon, M, 63y

Another barrier was the obstruction of patients' freewill, as they believed people have their own views about what is healthy or unhealthy. Some felt that they should only start such a conversation if the patient were to bring up the topic. HPs perceived lifestyle to be a low priority for the majority of survivors, although they acknowledged the cancer diagnosis as a "teachable moment" for them.

You have to be realistic as well. People will always make the wrong choices or choose to drink too much alcohol, or chose to smoke, or whatever, and that's freewill, isn't it, as well?

Breast Cancer Nurse, F, 46y

They don't see it as a priority. Their priority if much more about "What's happening with my cancer? Has it returned?". The financial issues, visa issues, all that sort of stuff. So, diet and lifestyle is somewhat down the line for a lot of patients.

Colorectal Cancer Nurse, F, 55y

I mean, a smoker, if they are determined not to give up there's absolutely nothing you can do about it. I mean, for some it's a real wake-up call and for some they really change their lifestyle. […] and then also sometimes people don't want to talk about it.

Prostate Cancer Physician, F, 54y

1.4 Socio-economic barriers to practise health behaviours

HP's perceived practical barriers for survivors included affordability of a healthy diet or gym subscription. Furthermore, perceived lack of social support and cultural variation were also reported as a barrier to practising health behaviours.

I think the difficulty is that depending on the demographics of patients you've got how practical it can be to either give the information or encourage people to follow the guidelines. As I say, our demographics are such that there's a lot of poverty within our catchment area. So that's often the biggest barrier to doing that. There's also… depending on cultural backgrounds, some of our

For peer review only - http://bmjopen.bmj.com/site/about/guidelines.xhtml

patients are not keen to take responsibility, they prefer the paternalistic approach. So that can be quite difficult. We also have a lot of patients who are very socially isolated, and are not inclined to go shopping, or cook for themselves.

Colorectal Cancer Nurse, F, 55y

2. HP-centred barriers to provision of lifestyle advice

2.1 Knowledge and attitudes towards evidence and guidelines for health behaviours

HPs tended to be aware only of general lifestyle guidelines, such as the Department of Health alcohol guidelines, rather than specific guidelines for cancer survivors. Others were not aware of any guidelines.

I've come across research papers about things like that [smoking and diet], but I haven't come across any guidelines.

Physiotherapist, F, 51y

Their attitude towards these general guidelines was positive but they based their advice only loosely on these. The advice tended to focus on either general health or controlling side effects and improving recovery. Only some HPs advised survivors that a healthy lifestyle might improve disease outcomes.

I do take the guidance into consideration but I do a bit more patient-centred approach, really.

Colorectal Cancer Nurse, F, 51y

So, I have always preached to patients that it's actually good to go our for a walk, get some fresh air, that sort of thing. But, obviously, more recently, there's been data both… for many of the polyp sort of cancers – whether it's breast or urological cancers – that getting physical exercise does improve outcomes. So, from the historical data to much more robust data, I'm pretty sold on the idea that just getting fresh air, going out for a walk, sensible exercise, not being… not heroic exercise, is good for the mind, good for the body, and good for physical recovery.

Prostate Cancer Physician, M, 49y

They also regarded the evidence on healthy lifestyles, cancer prevention, and cancer recurrence as not definitive. They perceived the evidence for physical activity on

For peer review only - http://bmjopen.bmj.com/site/about/guidelines.xhtml

cancer recurrence to be stronger than that available for any dietary factors; however, they were still advocates of a healthy diet for overall health. There were mixed views regarding the role of lifestyle in cancer recurrence, with only some professionals aware of this link and research findings.

> *Well, I suppose that it helps to reduce the risk of recurrence. A good healthy diet and to take a good… you know, exercise.*
>
> Breast Cancer Physician, M, 40y

> *I wouldn't say that diet and physical activity are related to cancer recurrence.*
>
> Colorectal Cancer Physician, F, 41y

The fact that lifestyle is one of multiple cancer risk factors made them less inclined to focus their consultations on it.

> *There's obviously other factors at work and at play and, although I encourage patients to take exercise and to eat a healthy diet, particularly with lots of veg, I don't want to be creating a guilt feeling that, if they do all of this, it will stop it coming back. And therefore, if it comes back it's because they didn't eat the right diet. So I think it is important, but there's so many other factors involved that I don't want to concentrate on this to the detriment of others.*
>
> Colorectal Cancer Physician, F, 44y

In contrast, evidence to back claims about the impact of behaviour change was seen as both important to facilitate change and as something that patients do not pay attention to.

> *Perhaps it would be helpful for patients to have some information that specifically says: 'We know that patients who stop smoking… We know that patients who are more active… We know that patients who keep within a healthy weight…' and then it has those percentages for reduced risk of recurrence, etc. […] perhaps it would motivate them.*
>
> Breast Cancer Nurse, F, 46y

HPs also mentioned that they could not quantify the effect of lifestyle changes post-treatment in terms of any outcome, given the lack of research data. This made their

For peer review only - http://bmjopen.bmj.com/site/about/guidelines.xhtml

argument weak and the conversation more challenging than those conversations focusing on medical treatments.

> *I can tell them if you take endocrine therapy, for survival, it would be improved by 10%. Chemotherapy, another 10%. So, 20% improvement. So there is some evidence for them for me to talk to the patient with the other treatments I'm offering, but I don't have anything for these [healthy lifestyle]. So that's another reason I won't be able to give them full information, that's when I say lack of information to give to the patients.*
>
> Breast Cancer Surgeon, M, 44y

2.2 Self-identification as the right person to provide lifestyle advice

While some felt it was their duty to empower survivors to lead a healthier lifestyle, others thought that they were not the right person to provide lifestyle advice, given their limited knowledge on the topic and other priorities during their short consultations.

> *And I think we have a duty to advise patients on quitting smoking and reducing alcohol intake and stuff.*
>
> Colorectal Cancer Nurse, F, 38y

> *I don't think it's necessarily my role to provide generic lifestyle advice to everybody, and unfortunately there isn't the time. I tend to focus on the people who have a problem, like fatigue, which I think would respond to exercise.*
>
> Prostate Cancer Physician, F, 34y

Some HPs thought their authority might help survivors take the advice more seriously, while others perceived their advice to be mostly ineffective.

> *I don't think that doctors necessarily are the best [to provide advice] unless they are felt to be a, sort of, place of authority for the patient.*
>
> Prostate Cancer Physician, F, 54y

> *There aren't many people I've advised to stop smoking who have ever done it. So I'm fairly sceptical about the value of my advice even though I persist with it.*
>
> Breast Cancer Physician, M, 64y

For peer review only

For peer review only - http://bmjopen.bmj.com/site/about/guidelines.xhtml

HPs perceived their own lack of adherence to lifestyle guidelines as both a barrier and a facilitator for providing advice; by not being a role model and by being able to relate to the challenge of behaviour change during the conversation with patients, respectively.

> *I don't have a BMI under 25, so I have no room to talk. Sometimes, though, a bit of 'I know how you feel' and a bit of feeling goes a long way. I know that one of my breast cancer nurses, who is very slim and very athletic, finds it far more difficult to talk to people about losing weight, because she doesn't feel she has anywhere to come from.*
>
> Breast Cancer Surgeon, F, 53y

2.3 Practical barriers to lifestyle advice provision

HPs regarded lack of time as a significant barrier for provision of lifestyle advice.

> *I don't have enough time to do it all properly and sensitively.*
>
> Colorectal Cancer Physician, F, 44y

They mentioned that they mostly provide verbal lifestyle advice due to a lack of other type of resources. Some tended to reiterate this advice in their patient letters, provide leaflets generated by their local multidisciplinary team, or refer people to existing relevant services for further support.

> *If some patients I think will benefit from a proper programme, then we give them this Living Well leaflet. If some patients who are saying they only have exercise and they are happy with it, then we don't given them that Living Well programme or healthy lifestyle leaflet. Apart from that, I don't have any other leaflets about diet or exercise as such, a specific how it improves their breast cancer survival. So in those questions I don't have nothing much to give them. And in my letter, like one line, I'll just mention to the patient the importance of maintaining a healthy weight, diet, and exercise.*
>
> Breast Cancer Surgeon, M, 44y

3. Optimal delivery of lifestyle advice

3.1 Tailored advice delivered throughout the cancer journey

For peer review only - http://bmjopen.bmj.com/site/about/guidelines.xhtml

HPs stressed that there is not a one-size-fit-all optimal format of lifestyle advice and were keen on providing choice of multiple formats, so that the patient could choose the one that fit best their individual needs and preferences. This was thought to enhance intervention adherence.

> *The level of intervention will depend on the patients, their background, the sort of feedback they give us to their motivation.*
>
> Prostate Cancer Physician, M, 49y

> *I think the best format is that somebody talks to the patient and asks them what they would want, and then plan the intervention accordingly rather than having one fitting for everybody, because that's not going to help.*
>
> Prostate Cancer Physician, F, 54y

The views on the optimal timing of providing advice were varied with some suggesting to intervene at diagnosis and others at the end of treatment. Some suggested that the optimal timing is patient-dependent. Others felt that the advice should be initiated at diagnosis and reiterated in each follow-up appointment.

> *It's something that should have been on the agenda all the way through and then [at the end of treatment] you're literally summarising, concluding, and then exploring if there's something they want to take over to another level.*
>
> Prostate Cancer Physician, M, 49y

HPs mentioned that a combination of verbal and written lifestyle information would be preferable to only verbal advice, as patients receive too much information during their cancer journey and they cannot take in everything. This was viewed as preferable to written advice alone as it could be made more relevant to the patient. HPs felt that all HPs should provide lifestyle advice.

> *If I just gave them a leaflet… I think it needs to be given in conjunction with a health professional or somebody who can talk them through it. If you just give them another leaflet, they're just likely to put it with their pack of other things they've got.*
>
> Physiotherapist, F, 51y

HPs in favour of advice using a face-to-face meeting with a HP felt that this meeting should be part of the general follow-up to boost attendance and acceptability. They mentioned that the meeting could enhance continuity of care post-treatment,

For peer review only - http://bmjopen.bmj.com/site/about/guidelines.xhtml

fostering personal contact with a professional aware of the patient's journey. For the survivors who would prefer the support of a group-based intervention, local community venues were preferred over hospital rooms. Online advice was deemed appropriate for technology-literate survivors.

> *A group intervention is definitely a positive thing for patients to get that peer support from other patients as well. I think it helps patients to feel that they can manage their condition better themselves and also with support from other patients rather than it just being medically led all the time, particularly if it's off site as well, if it's in other, perhaps, community settings as well.*
>
> Breast Cancer Nurse, F, 46y

> *I think online is probably the best thing, if patients have access and they're computer literate, because they can do it at their own time and at their own pace.*
>
> Colorectal Cancer Nurse, F, 55y

3.2 Small and achievable changes framed as part of their treatment regimen

HPs mentioned that survivors who need to change multiple behaviours might be overwhelmed by the process and suggested prioritising and tackling one or two behaviours at a time focusing on small realistic changes.

> *I'm aware that if you give people too much advice all at once, it's quite difficult for them to take it all in, so sometimes you are better off just focusing on one thing […] finding out which you think is the most risky of their behaviours and concentrate on that one.*
>
> Prostate Cancer Nurse, F, 53y

Some thought that framing the healthy lifestyle advice as part of their treatment would make it more engaging.

> *It would be nice to have some guidelines available, some specific booklet available about benefits of healthy diet and exercise, so we can give it to them as well, so they can follow it as a specific treatment regimen. [...] It is a treatment – healthy diet and exercise – and say it's a treatment. We should make them feel like that.*
>
> Breast Cancer Surgeon, M, 44y

For peer review only

I'd start telling them from the moment I see them with their diagnosis, because I want them to feel it's as important for them to undertake physical activity and eat a good diet and lose some weight as is their other treatments, like radiotherapy, and hormone blocking treatment. […] I tell them it will be part of their treatment so that they are prepared and accept it as normal.

Breast Cancer Surgeon, F, 41y

3.3 Cost-effectiveness for wide-scale implementation

Time and financial resources and proven cost-effectiveness were cited as barriers for wide-scale implementation of lifestyle advice. Lower need for resources indicated higher potential for implementation but HPs were unsure of measurable outcomes for one-off low-intensity lifestyle interventions, such as a leaflet or a phone call.

I think the patients would like the face-to-face. I suppose it just depends on what the resources are, as to who is doing it – is it a dietitian, is it a CNS, is it another organisation? So I think it depends on the manpower, what resources are available for a face-to-face. I think if it was extra on top of what we currently provide then we would struggle.

Colorectal Cancer Nurse, F, 54y

Integration is fine [for leaflet and online advice]. The question is: will it help?

Prostate Cancer Physician, F, 54y

Discussion

The current study reiterated that HPs' desire to provide lifestyle advice is not necessarily matched by knowledge and action. Reported barriers to provision included perceptions about survivor's behaviours, abilities and interest in advice, as well as concerns about HP's own abilities to provide effective advice without damaging their patient relationships. Nonetheless, HPs in this study were keen for most survivors to receive lifestyle advice throughout the cancer journey. Suggestions for this advice, included tailoring to individual patient needs and preferences, initiating small and achievable change goals, and framing recommendations as part of the patient's treatment regimen.

For peer review only - http://bmjopen.bmj.com/site/about/guidelines.xhtml

Previously reported barriers to provision of lifestyle advice include perception that patients lack interest in behaviour change, perception that behaviour change is not feasible or effective, lack of guidelines and confusion about the research findings, self-identification as not the right person to provide advice, potential for blaming the patient, lack of appropriate behaviour change support, and lack of time.[16 20 21] The current study confirmed the above barriers offering further insight, such as the perception that providing advice on alcohol and smoking is more challenging compared to diet and physical activity. Another insight was that the fear of blaming relates not only to cancer onset but also to cancer recurrence. The study also added to the list of barriers the potential loss of credibility and connection with survivors, the obstruction of free will, the importance of role modelling, and the challenge of addressing the co-existence of multiple suboptimal behaviours given practical barriers to implementing advice, such as affordability of a healthy diet. These factors could be taken into consideration in the development of programmes aiming to train HPs to effectively deliver lifestyle advice, as evidence suggests that even brief advice from HPs is effective for lifestyle behaviour change.[22-24]

Interestingly, the HPs' self-identification as not the right person to provide advice and their fear of losing the connection with their patients by providing them with continuous lifestyle advice, are not matched by the fact that survivors would actually welcome lifestyle advice from their oncology HPs.[13-15] Survivors' views partly support the perception of HPs that it is difficult to change engrained unhealthy lifelong habits. However, although there is little evidence that survivors make major changes in their health behaviours following a cancer diagnosis,[8 12] some report motivation to engage in behaviour change.[13 25] HP's therefore have an opportunity to capitalise on this and to provide lifestyle advice to survivors or signpost them to further support. These interventions should include social support, because this behaviour change technique was perceived to facilitate performing health behaviours by both HPs in the current study and survivors.[15]

However, HPs in this study did not mention reputable and long-standing lifestyle guidelines for cancer survivors.[11] One reason for the lack of awareness might be that these guidelines are based on the cancer prevention literature. However, the evidence base for their implementation post-diagnosis is growing, and they are

For peer review only - http://bmjopen.bmj.com/site/about/guidelines.xhtml

considered to be prudent advice for cancer survivors. The lack of dissemination of these guidelines should therefore be targeted with educational courses, conferences, and, training to enable HPs to provide evidence-based advice. Professional organisations might be pivotal in this process. Programmes to enhance oncology HPs knowledge on lifestyle guidelines and facilitate provision of advice are currently being piloted, and are showing promising potential.[26 27] These programmes should ensure not only adequate knowledge of the guidelines but also provide the tools and techniques to HPs to ensure they approach the topic of behaviour change in a supportive way with confidence and sensitivity, so that cancer survivors feel motivated and not guilty.

HPs in this study also indicated that the format of lifestyle advice should be tailored to individual survivors. Surveys, qualitative studies, and analysis of data from large behaviour change interventions are required to identify factors that predict cancer survivors' uptake and adherence to various types of lifestyle interventions. However, an analysis of three large home-based lifestyle interventions in cancer survivors did not identify any predictors of adherence to the different types of intervention.[28] To that end, quasi-experimental intervention designs (e.g. face-to-face vs. remote intervention) based on survivors' preferences for delivery format might better facilitate understanding of adherence and lead to effective personalised lifestyle support. Framing the intervention as part of the treatment regimen instead of health promotion advice could also be explored in future studies, with pilot studies indicating the effectiveness of physical activity rehabilitation on fitness levels.[29]

Strengths of the current study include the examination of HPs' views on key modifiable lifestyle behaviours from a sample of various HPs from across the UK. Although qualitative studies provide in-depth information, their findings cannot be generalised. It is possible that the participants of this study were generally interested in lifestyle topics, which might bias the responses towards a positive view on lifestyle. Furthermore, the views may not be representative of all oncology HPs. Further research could explore this, as HPs working with other cancers may be even less likely to give advice.

For peer review only - http://bmjopen.bmj.com/site/about/guidelines.xhtml

In conclusion, the findings of this study suggest that some, but not all, HPs specialising in breast, prostate and colorectal cancers provide lifestyle advice to their patients post-treatment. Those that provide advice describe providing generic guidance broadly based on healthy lifestyle recommendations for the general population, with attempts to tailor it to the individual patient. Additional education is needed to improve awareness of available guidelines for cancer survivors. Incorporation of the identified factors that influence the provision of lifestyle advice in the development of training programmes for HPs and lifestyle interventions for cancer survivors could increase the probability of effectiveness and in turn, improve outcomes for cancer survivors.

For peer review only

For peer review only - http://bmjopen.bmj.com/site/about/guidelines.xhtml

Acknowledgements

All authors were supported by funding from Cancer Research UK. SL was also funded by University College London and the Weight Concern Impact Award Studentship. RJB is currently supported by Yorkshire Cancer Research Academic Fellowship funding. The researchers are grateful to the health professionals that participated in the study.

Declaration of Conflicting Interests

The authors declare no conflict of interests.

Contributorship statement

RJB conceived the study and obtained the funding for the Impact Award Studentship. SL, RJB, AF, KW, and HC devised the interview protocol. SL conducted the interviews. SL, DAK, and RJB analysed the data with contribution from all authors. DAK drafted the manuscript. All authors critically revised, and approved the final version of the manuscript.

Data sharing

Anonymised interview transcripts can be obtained by the corresponding author on reasonable request.

For peer review only

For peer review only - http://bmjopen.bmj.com/site/about/guidelines.xhtml

References

1. Maddams J, Utley M, Moller H. Projections of cancer prevalence in the United Kingdom, 2010-2040. *Br J Cancer* 2012;107(7):1195-202. doi: 10.1038/bjc.2012.366
2. Quaresma M, Coleman MP, Rachet B. 40-year trends in an index of survival for all cancers combined and survival adjusted for age and sex for each cancer in England and Wales, 1971-2011: a population-based study. *Lancet* 2015;385(9974):1206-18. doi: 10.1016/S0140-6736(14)61396-9 [published Online First: 2014/12/07]
3. Keats MR, Cui Y, Grandy SA, et al. Cardiovascular disease and physical activity in adult cancer survivors: a nested, retrospective study from the Atlantic PATH cohort. *J Cancer Surviv* 2017;11(2):264-73. doi: 10.1007/s11764-016-0584-x [published Online First: 2016/11/18]
4. Duan W, Li S, Meng X, et al. Smoking and survival of breast cancer patients: A meta-analysis of cohort studies. *Breast* 2017;33:117-24. doi: 10.1016/j.breast.2017.03.012 [published Online First: 2017/04/04]
5. Schwedhelm C, Boeing H, Hoffmann G, et al. Effect of diet on mortality and cancer recurrence among cancer survivors: a systematic review and meta-analysis of cohort studies. *Nutr Rev* 2016;74(12):737-48. doi: 10.1093/nutrit/nuw045 [published Online First: 2016/11/20]
6. Stacey FG, James EL, Chapman K, et al. A systematic review and meta-analysis of social cognitive theory-based physical activity and/or nutrition behavior change interventions for cancer survivors. *J Cancer Surviv* 2015;9(2):305-38. doi: 10.1007/s11764-014-0413-z
7. Stolley M, Sheean P, Gerber B, et al. Efficacy of a Weight Loss Intervention for African American Breast Cancer Survivors. *J Clin Oncol* 2017:JCO2016719856. doi: 10.1200/JCO.2016.71.9856 [published Online First: 2017/06/20]
8. Rock CL, Doyle C, Demark-Wahnefried W, et al. Nutrition and physical activity guidelines for cancer survivors. *CA Cancer J Clin* 2012;62(4):243-74. doi: 10.3322/caac.21142
9. Schmitz KH, Courneya KS, Matthews C, et al. American College of Sports Medicine roundtable on exercise guidelines for cancer survivors. *Med Sci Sports Exerc* 2010;42(7):1409-26. doi: 10.1249/MSS.0b013e3181e0c112
10. WCRF. Continuous Update Project Report: Diet, Nutrition, Physical Activity, and Breast Cancer Survivors: World Cancer Research Fund International, 2014.
11. WCRF. Food, Nutrition, Physical Activity and the Prevention of Cancer: A Global Perspective. Washington, DC: World Cancer Research Fund / American Institute for Cancer Research, 2007.
12. Williams K, Steptoe A, Wardle J. Is a cancer diagnosis a trigger for health behaviour change? Findings from a prospective, population-based study. *Br J Cancer* 2013;108(11):2407-12. doi: 10.1038/bjc.2013.254
13. Beeken RJ, Williams K, Wardle J, et al. "What about diet?" A qualitative study of cancer survivors' views on diet and cancer and their sources of information. *Eur J Cancer Care (Engl)* 2016;25(5):774-83. doi: 10.1111/ecc.12529
14. Koutoukidis DA, Beeken RJ, Lopes S, et al. Attitudes, challenges, and needs about diet and physical activity in endometrial cancer survivors: a qualitative study. *Eur J Cancer Care (Engl)* 2016 doi: 10.1111/ecc.12531

For peer review only - http://bmjopen.bmj.com/site/about/guidelines.xhtml

15. Smith L, Croker H, Fisher A, et al. Cancer survivors' attitudes towards and knowledge of physical activity, sources of information, and barriers and facilitators of engagement: A qualitative study. *Eur J Cancer Care (Engl)* 2017;26(4) doi: 10.1111/ecc.12641 [published Online First: 2017/01/31]
16. Williams K, Beeken RJ, Fisher A, et al. Health professionals' provision of lifestyle advice in the oncology context in the United Kingdom. *Eur J Cancer Care (Engl)* 2015;24(4):522-30. doi: 10.1111/ecc.12305
17. Bengtsson M. How to plan and perform a qualitative study using content analysis. *NursingPlus Open* 2016;2(Supplement C):8-14. doi: https://doi.org/10.1016/j.npls.2016.01.001
18. Hsieh HF, Shannon SE. Three approaches to qualitative content analysis. *Qual Health Res* 2005;15(9):1277-88. doi: 10.1177/1049732305276687
19. Tong A, Sainsbury P, Craig J. Consolidated criteria for reporting qualitative research (COREQ): a 32-item checklist for interviews and focus groups. *Int J Qual Health Care* 2007;19(6):349-57. doi: 10.1093/intqhc/mzm042
20. Miles A, Simon A, Wardle J. Answering patient questions about the role lifestyle factors play in cancer onset and recurrence: what do health care professionals say? *J Health Psychol* 2010;15(2):291-8. doi: 10.1177/1359105309351245
21. Sutton E, Hackshaw-McGeagh LE, Aning J, et al. The provision of dietary and physical activity advice for men diagnosed with prostate cancer: a qualitative study of the experiences and views of health care professionals, patients and partners. *Cancer causes & control : CCC* 2017;28(4):319-29. doi: 10.1007/s10552-017-0861-7
22. NICE. Physical activity: brief advice for adults in primary care. In: Excellence NIfHaC, ed., 2013.
23. Jackson SE, Wardle J, Johnson F, et al. The impact of a health professional recommendation on weight loss attempts in overweight and obese British adults: a cross-sectional analysis. *BMJ Open* 2013;3(11):e003693. doi: 10.1136/bmjopen-2013-003693
24. Fisher A, Williams K, Beeken R, et al. Recall of physical activity advice was associated with higher levels of physical activity in colorectal cancer patients. *BMJ Open* 2015;5(4):e006853. doi: 10.1136/bmjopen-2014-006853
25. Anderson AS, Steele R, Coyle J. Lifestyle issues for colorectal cancer survivors--perceived needs, beliefs and opportunities. *Support Care Cancer* 2013;21(1):35-42. doi: 10.1007/s00520-012-1487-7
26. Webb J, Hall J, Hall K, et al. Increasing the frequency of physical activity very brief advice by nurses to cancer patients. A mixed methods feasibility study of a training intervention. *Public health* 2016;139:121-33. doi: 10.1016/j.puhe.2016.05.015
27. Murphy J, Worswick L, Pulman A, et al. Translating research into practice: evaluation of an e-learning resource for health care professionals to provide nutrition advice and support for cancer survivors. *Nurse Educ Today* 2015;35(1):271-6. doi: 10.1016/j.nedt.2014.05.009 [published Online First: 2014/06/28]
28. Adams RN, Mosher CE, Blair CK, et al. Cancer survivors' uptake and adherence in diet and exercise intervention trials: an integrative data analysis. *Cancer* 2015;121(1):77-83. doi: 10.1002/cncr.28978
29. West MA, Loughney L, Lythgoe D, et al. Effect of prehabilitation on objectively measured physical fitness after neoadjuvant treatment in preoperative rectal

For peer review only - http://bmjopen.bmj.com/site/about/guidelines.xhtml

639 cancer patients: a blinded interventional pilot study. *British journal of*
640 *anaesthesia* 2015;114(2):244-51. doi: 10.1093/bja/aeu318
641

For peer review only

Supplementary material 1: Interview Topic Guide

	Topic	Question	Prompt
1.	Introductions / background	Brief introductions; get verbal consent to tape-record the interview; remind the aims of study, check length of interview/audio-recorded.	
2.	Health beliefs	What are your views on the role of diet and physical activity in the recovery from [breast/prostate/colorectal] cancer?<br>What are your views on the role of diet and physical activity in cancer recurrence?	
3.	Awareness of lifestyle guidelines for cancer survivors	Are you familiar with any guidelines specifically for cancer patients for any of the following lifestyle topics?<br>You said that you were aware of “X, Y, Z” guidelines. Can you please tell me what you know (or what you remember) about each of these guidelines?	Prompt<br>- PA<br>- Diet<br>- Weight management<br>- Smoking<br>- Alcohol
		What are your views on these guidelines? What do you think about them?	Prompt<br>- Liking<br>- Realistic<br>- Reliable
		How did you find out about them?<br>You also said that you were not aware of the “K, M” guidelines. Why do you think that you did not come across these guidelines?<br>What were the barriers for you not getting this information?	
3.	Barriers for provision of lifestyle advice	Thinking of all your patients who have completed primary treatment for cancer, do you give your patients advice on any lifestyle topics?	Prompt<br>- PA<br>- Diet<br>- Weight management<br>- Smoking<br>- Alcohol
		You said that you give advice on “X lifestyle topic” to “X%” of your patients. What kind of advice do you tend to give?	Prompt<br>- Verbal

For peer review only

Topic	Question	Prompt
		- Written<br>- Website referral<br>- HP referral<br>- When / Why this specific format<br>- Guidelines based
	Why do you think that you provide advice on this to [so many / only to x%] of your patients?<br>What prevents you from providing this type of advice to more patients?<br>What are the reasons why you don't usually provide lifestyle advice?	Prompt<br>- Verbal<br>- Written<br>- Website referral<br>- HP referral
	For each lifestyle topic that professional does NOT provide any advice, ask: Is there any particular reason why you have not given advice on "X lifestyle topic" to any of your patients?<br>What are the barriers preventing you to provide this advice?	Prompt<br>- Guidelines
	On the survey, we asked you about a number of specific barriers preventing you to provide lifestyle advice for your patients. You mentioned that "X" and "Y" were the main barriers for you. Can you tell me more about these barriers? Why these prevent you from providing lifestyle advice?<br>Are there any other barriers?	
5. Content Expectations	Have any of your cancer patients ask you information about lifestyle?<br><br>What type of information do they usually ask for?<br>How often do they ask you about lifestyle information? Why?<br>Is there any other type of information they should be getting? Why do you think it is important they get that information?<br>Is there anything you would avoid in telling them? Why?	

For peer review only

Topic	Question	Prompt
	Have any of the relatives, family members, friends or carers of your patients asked you information about lifestyle?<br>What type of information do they usually ask for?<br>How often do they ask you about lifestyle information? Why?<br>Is there any other type of information they should be getting? Why do you think it is important they get that information?<br>Is there anything you would avoid in telling relatives, friends or carers? Why?	
6. Delivery	We are planning to develop some materials to provide information on diet and physical activity for people who have had cancer treatment.<br>When would it be the best time for health professionals to provide information on healthy eating and physical activity to colorectal cancer patients? Why?<br>Do you think patients would be open to receive this type of information at that time? Why?<br>Who should provide them information about healthy eating and physical activity? Why?<br>In what format do you think that it would be better to provide information about diet and physical activity, for colorectal cancer patients? Why?	
	What do you think about a leaflet?<br>What do you think about a leaflet along with a telephone call from a HP?<br>What do you think about a leaflet along with a face-to-face contact with a HP?<br>What do you think about a leaflet along with online information?<br>What do you think about a self-help book with specific information for cancer survivors?<br>What do you think about a group intervention designed for cancer survivors with about 8 sessions, providing more detailed lifestyle information?<br>We have discussed about several options and formats to provide lifestyle information. Balancing between what would be better for HPs, for patients and feasibility of implementation; what do you think would be the best format to provide lifestyle advice?	Prompt<br>- Usefulness<br>- Receipt<br>- Implementation
	From your perspective, what are the issues or barriers to take into account that some [breast/prostate/colorectal] cancer patients might face in trying to change their	Prompt<br>- Mood

For peer review only

Topic	Question	Prompt
	lifestyle?	- Mental health<br>- Physical restrictions<br>- Visual problems
7. Ending	Do you have anything else to add, any comments or any questions?	

For peer review only

Supplementary material 2 – Interviews NVIVO Coding Framework

(1) Health Beliefs

- Beliefs about role of diet in cancer (incl. cancer recurrence)
- Beliefs about role of exercise in cancer (incl. cancer recurrence)
- Beliefs about role of alcohol in cancer (incl. cancer recurrence)
- Beliefs about role of smoking in cancer (incl. cancer recurrence)
- Health beliefs (not lifestyle)

(2) Awareness of lifestyle guidelines for cancer survivors

- Aware of guidelines (& description)
- Not aware of guidelines (& description)
- Views on guidelines
- Barriers to accessing guidelines (incl. not aware)
- How found out about guidelines

(3) Provision of lifestyle advice

- Advice given (diet)
- Advice given (exercise)
- Advice given (smoking)
- Advice given (alcohol)
- Advice given (weight)
- Advice given and requested by Relatives
- Advice would like to get on other topics
- Barriers to provision of advice
- Reason for provision of advice to a HIGH number of pts
- Reason for provision of advice to a LOW number of pts

(4) Format

- Timing
  - Timing HP has provided lifestyle advice
  - Preferred timing for provision of lifestyle advice
- Source

For peer review only

Mode of delivery (all negative, incl. leaflet, self-help book and group)

Ideal Mode of delivery

Mode of delivery of information provided (incl verbally, referral, web, leaflet, self help and group)

Format Group

Format Verbally

Format Digital

Format Written (All)

Leaflet only

Leaflet + telephone call

Leaflet + face-to-face contact

Leaflet + online information

Self-Help book

(5) Barriers to attending lifestyle interventions - internal & external

(6) Pathway issues

Leaflet only

Leaflet + telephone call

Leaflet + face to face contact

Leaflet + online information

Self-Help Book

Group intervention

Other Pathway factors

For peer review only

COREQ (COnsolidated criteria for REporting Qualitative research) Checklist

A checklist of items that should be included in reports of qualitative research. You must report the page number in your manuscript where you consider each of the items listed in this checklist. If you have not included this information, either revise your manuscript accordingly before submitting or note N/A.

Topic	Item No.	Guide Questions/Description	Reported on Page No.
**Domain 1: Research team and reflexivity**			
Personal characteristics			
Interviewer/facilitator	1	Which author/s conducted the interview or focus group?	5
Credentials	2	What were the researcher's credentials? E.g. PhD, MD	5
Occupation	3	What was their occupation at the time of the study?	5
Gender	4	Was the researcher male or female?	5
Experience and training	5	What experience or training did the researcher have?	5
Relationship with participants			
Relationship established	6	Was a relationship established prior to study commencement?	5
Participant knowledge of the interviewer	7	What did the participants know about the researcher? e.g. personal goals, reasons for doing the research	5
Interviewer characteristics	8	What characteristics were reported about the inter viewer/facilitator? e.g. Bias, assumptions, reasons and interests in the research topic	5
**Domain 2: Study design**			
Theoretical framework			
Methodological orientation and Theory	9	What methodological orientation was stated to underpin the study? e.g. grounded theory, discourse analysis, ethnography, phenomenology, content analysis	5
Participant selection			
Sampling	10	How were participants selected? e.g. purposive, convenience, consecutive, snowball	5
Method of approach	11	How were participants approached? e.g. face-to-face, telephone, mail, email	5
Sample size	12	How many participants were in the study?	5-6
Non-participation	13	How many people refused to participate or dropped out? Reasons?	5-6
Setting			
Setting of data collection	14	Where was the data collected? e.g. home, clinic, workplace	5
Presence of non-participants	15	Was anyone else present besides the participants and researchers?	N/A
Description of sample	16	What are the important characteristics of the sample? e.g. demographic data, date	6-7
Data collection			
Interview guide	17	Were questions, prompts, guides provided by the authors? Was it pilot tested?	5
Repeat interviews	18	Were repeat inter views carried out? If yes, how many?	N/A
Audio/visual recording	19	Did the research use audio or visual recording to collect the data?	5
Field notes	20	Were field notes made during and/or after the inter view or focus group?	5
Duration	21	What was the duration of the inter views or focus group?	5
Data saturation	22	Was data saturation discussed?	N/A
Transcripts returned	23	Were transcripts returned to participants for comment and/or	N/A

For peer review only

Topic	Item No.	Guide Questions/Description	Reported on Page No.
		correction?	
**Domain 3: analysis and findings**			
Data analysis			
Number of data coders	24	How many data coders coded the data?	5
Description of the coding tree	25	Did authors provide a description of the coding tree?	Appendix 2
Derivation of themes	26	Were themes identified in advance or derived from the data?	5-6
Software	27	What software, if applicable, was used to manage the data?	5
Participant checking	28	Did participants provide feedback on the findings?	N/A
Reporting			
Quotations presented	29	Were participant quotations presented to illustrate the themes/findings? Was each quotation identified? e.g. participant number	7-18
Data and findings consistent	30	Was there consistency between the data presented and the findings?	7-18
Clarity of major themes	31	Were major themes clearly presented in the findings?	7-18
Clarity of minor themes	32	Is there a description of diverse cases or discussion of minor themes?	7-18

Developed from: Tong A, Sainsbury P, Craig J. Consolidated criteria for reporting qualitative research (COREQ): a 32-item checklist for interviews and focus groups. *International Journal for Quality in Health Care*. 2007. Volume 19, Number 6: pp. 349 – 357

Once you have completed this checklist, please save a copy and upload it as part of your submission. DO NOT include this checklist as part of the main manuscript document. It must be uploaded as a separate file.

For peer review only

BMJ Open

Lifestyle advice to cancer survivors: a qualitative study on the perspectives of health professionals

Journal:	*BMJ Open*
Manuscript ID	bmjopen-2017-020313.R2
Article Type:	Research
Date Submitted by the Author:	05-Feb-2018

Complete List of Authors:	Koutoukidis, Dimitrios; University College London, Behavioural Science and Health; University of Oxford, Nuffield Department of Primary Care Health Sciences<br>Lopes, Sonia; University College London, Behavioural Science and Health<br>Fisher, Abigail; University College London, Behavioural Science and Health<br>Williams, Kate; University College London, Behavioural Science and Health<br>Croker, Helen; University College London, Behavioural Science and Health<br>Beeken, Rebecca; University College London, Behavioural Science and Health; University of Leeds Leeds Institute of Health Sciences

**Primary Subject Heading**:	Oncology
Secondary Subject Heading:	Qualitative research
Keywords:	health professionals, cancer survivors, lifestyle, guidelines, interventions

SCHOLARONE™ Manuscripts

For peer review only - http://bmjopen.bmj.com/site/about/guidelines.xhtml

Lifestyle advice to cancer survivors: a qualitative study on the perspectives of health professionals

Dimitrios A. Koutoukidis[1,2¶], Sonia Lopes[1¶], Abigail Fisher[1], Kate Williams[1], Helen Croker[1], Rebecca J. Beeken[1,3]

[1]Department of Behavioural Science and Health, University College London, London, UK

[2]Nuffield Department of Primary Care Health Sciences, University of Oxford, Oxford, UK

[3]Leeds Institute of Health Sciences, University of Leeds, Leeds, UK

[¶]Contributed equally

Corresponding Author:

Dr Rebecca Beeken

Leeds Institute of Health Sciences

Level 10, Worsley Building,

Clarendon Way,

Leeds, LS2 9NL Tel. +44 (0)113 343 3228 E-mail: r.beeken@leeds.ac.uk

For peer review only - http://bmjopen.bmj.com/site/about/guidelines.xhtml

Abstract

Objectives: Adoption of healthy lifestyle behaviours has shown promising effectiveness in reducing the high morbidity burden of cancer survivors. Health professionals (HPs) are well suited to provide lifestyle advice but few survivors report receiving guidance from them. This study aimed to explore HPs' perspective of lifestyle advice (on heathy eating, physical activity, smoking, and alcohol) for cancer survivors.

Design: In-depth semi-structured qualitative interviews were conducted by telephone or face-to-face. Data were analysed using qualitative content analysis.

Setting and participants: Twenty-one UK HPs working in secondary care with breast, prostate, or colorectal cancer survivors were interviewed.

Results: The over-arching theme was that HPs' desire to provide lifestyle advice was not necessarily matched by knowledge and action. Three main themes were identified: 1) survivorship-centred barriers to provision, 2) HP-centred barriers to provision, and 3) optimal delivery of lifestyle advice. Results suggested that HPs' perceptions of survivors' current status of practising health behaviours, their perceived socio-economic barriers and ability to practise health behaviours, and HPs' fear for potential loss of connection with the patient influenced provision of lifestyle advice. Further factors included HPs' knowledge of healthy lifestyle guidelines, feeling that they were not the 'right person' to provide advice, and lack of time and resources. HPs stressed that the optimal delivery of lifestyle advice should (a) be tailored to the individual and delivered throughout the cancer journey, (b) be focused on small and achievable changes framed as part of their treatment regimen, and (c) be cost-effective for wide-scale implementation.

Conclusions: Incorporation of the identified barriers when developing HP training programmes and lifestyle interventions could increase the probability of successful behaviour change and, thus, improved outcomes for cancer survivors.

Keywords: health professionals, cancer survivors, lifestyle, guidelines, interventions

For peer review only - http://bmjopen.bmj.com/site/about/guidelines.xhtml

Strengths and limitations of the study

- The study comprehensively examined the views of health professionals, working with breast, prostate, and colorectal cancer survivors, on key modifiable lifestyle behaviours.
- Health professionals had diverse professional backgrounds, including nursing, oncology, surgery, and allied health professions.
- It is possible that the participants were generally interested in lifestyle topics, which might bias the responses towards a positive view on lifestyle.
- Although qualitative studies provide in-depth information, their findings cannot be generalised.

For peer review only

Introduction

There are 2.5 million cancer survivors living in the UK.[1] The increasing number of cancer survivors and their higher morbidity burden compared to the general population means strategies are needed to improve survivorship outcomes.[2 3] Adoption of key modifiable lifestyle behaviours (i.e. not smoking or drinking alcohol, eating a healthy diet, engaging in sufficient physical activity, and weight management) are potential strategies. There is a growing body of evidence that suggests these health behaviours are positively associated with improved outcomes in cancer survivors.[4 5] Furthermore, behavioural lifestyle interventions in this population have shown promising effectiveness.[6 7]

There are now lifestyle guidelines for cancer survivors, advocating smoking cessation, moderate alcohol consumption (if any), a healthy balanced diet, daily physical activity, and maintenance of a healthy weight.[8-11] Although surveys suggest that few cancer survivors are meeting these guidelines,[12] qualitative data suggest that a cancer diagnosis may promote motivation for behaviour change and that survivors are keen to receive healthy lifestyle advice, ideally from a credible source.[13 14] Health professionals (HPs) are well positioned to provide healthy lifestyle advice and, thus, help cancer survivors improve their lifestyle behaviours. However, few survivors report receiving lifestyle advice from their HPs.[13 15]

We previously conducted a survey with HPs suggesting that 36% of HPs were not aware of any lifestyle guidelines for cancer survivors.[16] Although most reported providing some lifestyle advice, a number of barriers to provision were also cited, including lack of guidelines, belief that advice would not change behaviour or affect outcomes, belief that they are not the right person to provide advice, lack of time, lack of patient interest, fear that they would offend the patient, and cultural differences. These highlighted the potential for an intervention to help HPs provide evidence-based advice.

The aim of this qualitative study was to explore in greater depth HPs' views on the provision of lifestyle advice to patients with a cancer diagnosis.

Methods

For peer review only - http://bmjopen.bmj.com/site/about/guidelines.xhtml

Procedure and Participants

Eligible participants were UK-based HPs (including surgeons, physicians, nurses and allied HPs) caring for patients diagnosed with the most common lifestyle-related cancers (breast, prostate, or colorectal cancer). They had provided contact details following their participation in an online survey on lifestyle guidelines for cancer survivors and agreed to be contacted for future research.[16] HPs were invited via e-mail to participate in either a telephone (n=20) or an individual face-to-face (n=1) interview at University College London. Participants provided signed informed consent. Ethical approval was granted by the UCL Research Ethics Committee (project ID: 4456/001).

Interviews

Between October 2014 and January 2015, an experienced female research psychologist with interest in cancer survivorship (SL, MSc) interviewed the HPs. The interviewer had no previous relationship with the study participants. Participants were aware that the study was part of the interviewer's research degree. Each interview lasted approximately 40 minutes (range: 17-74 minutes). The interview followed a semi-structured guide (Supplementary material 1). The guide followed up on HPs' individual survey responses about the provision of advice to patients who had completed treatment, and used open-ended questions and prompts to explore in-depth HPs' awareness of survivorship lifestyle guidelines, their barriers to provision of lifestyle advice, and their views on various formats for lifestyle interventions. Interviews were audio-recorded, anonymised, and transcribed verbatim. The researcher listened to the tapes to verify them for accuracy. Field notes were not taken as all but one interview took place over the phone, meaning information could not be collected on setting, appearance or nonverbal behaviours of participants.

Analysis

Interview transcripts were analysed using qualitative content analysis[17] in NVivo version 10. Content analysis was chosen as the main aim of the study was to describe HPs views as opposed to developing theory or exploring the lived experience[18]. Following familiarisation with the data, meaning units and codes were generated using an inductive approach. SL coded the transcripts derived the coding list (Supplementary material 2) in an iterative process with RJB. An independent

For peer review only - http://bmjopen.bmj.com/site/about/guidelines.xhtml

reviewer with a dietetics background (DAK) coded 20% of interviews against the coding list. Inter-rater reliability was high (mean kappa=0.92) and minor coding differences were resolved by discussion. Categories and themes were compiled with a manifest analysis using an iterative process. This ensured internal homogeneity and external heterogeneity of the themes and categories. The completed COREQ checklist on standard reporting guidelines is available in Supplementary material 3.[19]

Results

Sample Characteristics

Fifty-two of the 336 survey participants were eligible for this study. Twenty-one (40.4%) were interviewed and 31 either did not respond or were unwilling to take part. Table 1 shows the sample characteristics of the study in relation to the survey. Most participants were female and aged between 46 and 55 years. Their professional role varied and their work location spanned across the UK.

For peer review only

Table 1: Sample characteristics in relation to the previous survey

	Interview participants n=21	Survey participants n=336[1]
	% (n)	% (n)
Gender		
Female	76 (16)	81 (272)
Male	24 (5)	19 (64)
Age *(in years)*		
26-35	5 (1)	12 (41)
36-45	38 (8)	33 (110)
46-55	48 (10)	42 (141)
56-65	10 (2)	13 (42)
Profession		
Nurse	43 (9)	55 (126)
Physician	33 (7)	21 (48)
Surgeon	19 (4)	13 (31)
Allied HP (physiotherapist)	5 (1)	11 (26)
Cancer Specialism		
Breast	43 (9)	25 (54)
Colorectal	38 (8)	15 (32)
Prostate	19 (4)	10 (21)
Region of Work		
North West England	38 (8)	-

For peer review only - http://bmjopen.bmj.com/site/about/guidelines.xhtml

London	24 (5)	-
South West England	10 (2)	-
South East England	10 (2)	-
Wales	10 (2)	-
East of England	5 (1)	-
Scotland	5 (1)	-

[1] Data from Williams et al 2015. % may not total 100% due to rounding.

Themes

The overarching theme arising from the interviews was that HPs' desire to provide lifestyle advice was not necessarily matched by knowledge and action. Three main themes were identified: 1) HP's perceptions of survivor-centred barriers to provision of lifestyle advice, 2) HP-centred barriers to provision of lifestyle advice, and 3) optimal delivery of lifestyle advice.

1 HPs' perceptions of survivor-centred barriers to provision of lifestyle advice

1.1. Perceptions of survivors' current health behaviours

HPs felt that lifestyle behaviour change was particularly challenging for cancer survivors, due to ingrained lifelong habits and sustainability of achieved changes.

> *Well, lifestyle is very much a matter of habit, and breaking habits is always difficult for all of us.*
>
> Breast Cancer Surgeon, M, 63y

Co-existence of multiple sub-optimal behaviours was regarded as a barrier to provision of lifestyle advice.

> *It is sometimes difficult when people just do seem to have all the vices, so they are heavy smokers, they are not just overweight, they are really obese, they obviously take no exercise and they are heavy smokers. It's very difficult to know sometimes where to start.*
>
> Breast Cancer Surgeon, F, 53y

Alcohol and tobacco were regarded as sensitive topics and it was perceived hard to assess consumption levels. Therefore, this was only addressed if there were evident indications of a problem or if the topic was raised by relatives.

> *[…] when it comes to alcohol it can be quite tricky. Sometimes we'll have relatives who will say, 'Are you aware this person is*

For peer review only

For peer review only - http://bmjopen.bmj.com/site/about/guidelines.xhtml

> *drinking?' particularly if they are on chemotherapy. And the side effects of chemotherapy, and then to add alcohol onto it. I think that's a more difficult area to try and challenge people on.*
>
> Colorectal Cancer Nurse, F, 54y

They felt that low mood and distress were also barriers for implementation of the provided advice.

> *If someone is depressed or has low mood, for whatever reason, everything is harder.*
>
> Breast Cancer Nurse, F, 40y

1.2 HPs' perception of survivors' ability to perform health behaviours

The lifestyle advice provided was mostly tailored to the individual patient. Frailty and physical inability to perform physical activity significantly hindered the HPs' inclination to provide physical activity advice. Tolerability of a high-fibre diet due to treatment-related bowel changes was also considered a barrier for provision of advice on healthy eating.

> *If somebody is frail and unwell generally, either because of their cancer or because of other medical problems, then I think advising them about diet and exercise and things isn't necessarily helpful for them.*
>
> Prostate Cancer Physician, F, 34y

> *I think some of our patients have got possibly some degree of stenosis, and actually saying to them to have a lot of fruit and fibre in their diet is wrong information. So, I think it has to be tailored to the individual, really.*
>
> Colorectal Cancer Nurse, F, 54y

1.3 Potential loss of connection with the patient

Fear of blaming the patient resulted in HPs taking a subtle rather than a strong approach to providing lifestyle advice. HPs did not want to make people feel guilty about their previous lifestyle or even create future blame if the patient did not subsequently change their lifestyle and the cancer reoccurred. They mentioned a potential loss of credibility and connection with their patients if they were to insist on lifestyle change.

For peer review only - http://bmjopen.bmj.com/site/about/guidelines.xhtml

I need to keep credibility with them as well, and if I start making demands of them which they really can't possibly keep then I kind of lose my own connection with these people.

Breast Cancer Surgeon, M, 63y

Another barrier was the obstruction of patients' freewill, as they believed people have their own views about what is healthy or unhealthy. Some felt that they should only start such a conversation if the patient were to bring up the topic. HPs perceived lifestyle to be a low priority for the majority of survivors, although they acknowledged the cancer diagnosis as a "teachable moment" for them.

You have to be realistic as well. People will always make the wrong choices or choose to drink too much alcohol, or chose to smoke, or whatever, and that's freewill, isn't it, as well?

Breast Cancer Nurse, F, 46y

They don't see it as a priority. Their priority if much more about "What's happening with my cancer? Has it returned?". The financial issues, visa issues, all that sort of stuff. So, diet and lifestyle is somewhat down the line for a lot of patients.

Colorectal Cancer Nurse, F, 55y

I mean, a smoker, if they are determined not to give up there's absolutely nothing you can do about it. I mean, for some it's a real wake-up call and for some they really change their lifestyle. […] and then also sometimes people don't want to talk about it.

Prostate Cancer Physician, F, 54y

1.4 Socio-economic barriers to practise health behaviours

HP's perceived practical barriers for survivors included affordability of a healthy diet or gym subscription. Furthermore, perceived lack of social support and cultural variation were also reported as a barrier to practising health behaviours.

I think the difficulty is that depending on the demographics of patients you've got how practical it can be to either give the information or encourage people to follow the guidelines. As I say, our demographics are such that there's a lot of poverty within our catchment area. So that's often the biggest barrier to doing that. There's also… depending on cultural backgrounds, some of our

For peer review only - http://bmjopen.bmj.com/site/about/guidelines.xhtml

patients are not keen to take responsibility, they prefer the paternalistic approach. So that can be quite difficult. We also have a lot of patients who are very socially isolated, and are not inclined to go shopping, or cook for themselves.

Colorectal Cancer Nurse, F, 55y

2. HP-centred barriers to provision of lifestyle advice

2.1 Knowledge and attitudes towards evidence and guidelines for health behaviours

HPs tended to be aware only of general lifestyle guidelines, such as the Department of Health alcohol guidelines, rather than specific guidelines for cancer survivors. Others were not aware of any guidelines.

I've come across research papers about things like that [smoking and diet], but I haven't come across any guidelines.

Physiotherapist, F, 51y

Their attitude towards these general guidelines was positive but they based their advice only loosely on these. The advice tended to focus on either general health or controlling side effects and improving recovery. Only some HPs advised survivors that a healthy lifestyle might improve disease outcomes.

I do take the guidance into consideration but I do a bit more patient-centred approach, really.

Colorectal Cancer Nurse, F, 51y

So, I have always preached to patients that it's actually good to go our for a walk, get some fresh air, that sort of thing. But, obviously, more recently, there's been data both… for many of the polyp sort of cancers – whether it's breast or urological cancers – that getting physical exercise does improve outcomes. So, from the historical data to much more robust data, I'm pretty sold on the idea that just getting fresh air, going out for a walk, sensible exercise, not being… not heroic exercise, is good for the mind, good for the body, and good for physical recovery.

Prostate Cancer Physician, M, 49y

They also regarded the evidence on healthy lifestyles, cancer prevention, and cancer recurrence as not definitive. They perceived the evidence for physical activity on

For peer review only - http://bmjopen.bmj.com/site/about/guidelines.xhtml

cancer recurrence to be stronger than that available for any dietary factors; however, they were still advocates of a healthy diet for overall health. There were mixed views regarding the role of lifestyle in cancer recurrence, with only some professionals aware of this link and research findings.

> *Well, I suppose that it helps to reduce the risk of recurrence. A good healthy diet and to take a good… you know, exercise.*
>
> Breast Cancer Physician, M, 40y

> *I wouldn't say that diet and physical activity are related to cancer recurrence.*
>
> Colorectal Cancer Physician, F, 41y

The fact that lifestyle is one of multiple cancer risk factors made them less inclined to focus their consultations on it.

> *There's obviously other factors at work and at play and, although I encourage patients to take exercise and to eat a healthy diet, particularly with lots of veg, I don't want to be creating a guilt feeling that, if they do all of this, it will stop it coming back. And therefore, if it comes back it's because they didn't eat the right diet. So I think it is important, but there's so many other factors involved that I don't want to concentrate on this to the detriment of others.*
>
> Colorectal Cancer Physician, F, 44y

In contrast, evidence to back claims about the impact of behaviour change was seen as both important to facilitate change and as something that patients do not pay attention to.

> *Perhaps it would be helpful for patients to have some information that specifically says: 'We know that patients who stop smoking… We know that patients who are more active… We know that patients who keep within a healthy weight…' and then it has those percentages for reduced risk of recurrence, etc. […] perhaps it would motivate them.*
>
> Breast Cancer Nurse, F, 46y

HPs also mentioned that they could not quantify the effect of lifestyle changes post-treatment in terms of any outcome, given the lack of research data. This made their

For peer review only - http://bmjopen.bmj.com/site/about/guidelines.xhtml

argument weak and the conversation more challenging than those conversations focusing on medical treatments.

> *I can tell them if you take endocrine therapy, for survival, it would be improved by 10%. Chemotherapy, another 10%. So, 20% improvement. So there is some evidence for them for me to talk to the patient with the other treatments I'm offering, but I don't have anything for these [healthy lifestyle]. So that's another reason I won't be able to give them full information, that's when I say lack of information to give to the patients.*
>
> Breast Cancer Surgeon, M, 44y

2.2 Self-identification as the right person to provide lifestyle advice

While some felt it was their duty to empower survivors to lead a healthier lifestyle, others thought that they were not the right person to provide lifestyle advice, given their limited knowledge on the topic and other priorities during their short consultations.

> *And I think we have a duty to advise patients on quitting smoking and reducing alcohol intake and stuff.*
>
> Colorectal Cancer Nurse, F, 38y

> *I don't think it's necessarily my role to provide generic lifestyle advice to everybody, and unfortunately there isn't the time. I tend to focus on the people who have a problem, like fatigue, which I think would respond to exercise.*
>
> Prostate Cancer Physician, F, 34y

Some HPs thought their authority might help survivors take the advice more seriously, while others perceived their advice to be mostly ineffective.

> *I don't think that doctors necessarily are the best [to provide advice] unless they are felt to be a, sort of, place of authority for the patient.*
>
> Prostate Cancer Physician, F, 54y

> *There aren't many people I've advised to stop smoking who have ever done it. So I'm fairly sceptical about the value of my advice even though I persist with it.*
>
> Breast Cancer Physician, M, 64y

For peer review only

HPs perceived their own lack of adherence to lifestyle guidelines as both a barrier and a facilitator for providing advice; by not being a role model and by being able to relate to the challenge of behaviour change during the conversation with patients, respectively.

> *I don't have a BMI under 25, so I have no room to talk. Sometimes, though, a bit of 'I know how you feel' and a bit of feeling goes a long way. I know that one of my breast cancer nurses, who is very slim and very athletic, finds it far more difficult to talk to people about losing weight, because she doesn't feel she has anywhere to come from.*
>
> Breast Cancer Surgeon, F, 53y

2.3 Practical barriers to lifestyle advice provision

HPs regarded lack of time as a significant barrier for provision of lifestyle advice.

> *I don't have enough time to do it all properly and sensitively.*
>
> Colorectal Cancer Physician, F, 44y

They mentioned that they mostly provide verbal lifestyle advice due to a lack of other type of resources. Some tended to reiterate this advice in their patient letters, provide leaflets generated by their local multidisciplinary team, or refer people to existing relevant services for further support.

> *If some patients I think will benefit from a proper programme, then we give them this Living Well leaflet. If some patients who are saying they only have exercise and they are happy with it, then we don't given them that Living Well programme or healthy lifestyle leaflet. Apart from that, I don't have any other leaflets about diet or exercise as such, a specific how it improves their breast cancer survival. So in those questions I don't have nothing much to give them. And in my letter, like one line, I'll just mention to the patient the importance of maintaining a healthy weight, diet, and exercise.*
>
> Breast Cancer Surgeon, M, 44y

3. Optimal delivery of lifestyle advice

3.1 Tailored advice delivered throughout the cancer journey

For peer review only - http://bmjopen.bmj.com/site/about/guidelines.xhtml

HPs stressed that there is not a one-size-fit-all optimal format of lifestyle advice and were keen on providing choice of multiple formats, so that the patient could choose the one that fit best their individual needs and preferences. This was thought to enhance intervention adherence.

> *The level of intervention will depend on the patients, their background, the sort of feedback they give us to their motivation.*
>
> Prostate Cancer Physician, M, 49y

> *I think the best format is that somebody talks to the patient and asks them what they would want, and then plan the intervention accordingly rather than having one fitting for everybody, because that's not going to help.*
>
> Prostate Cancer Physician, F, 54y

The views on the optimal timing of providing advice were varied with some suggesting to intervene at diagnosis and others at the end of treatment. Some suggested that the optimal timing is patient-dependent. Others felt that the advice should be initiated at diagnosis and reiterated in each follow-up appointment.

> *It's something that should have been on the agenda all the way through and then [at the end of treatment] you're literally summarising, concluding, and then exploring if there's something they want to take over to another level.*
>
> Prostate Cancer Physician, M, 49y

HPs mentioned that a combination of verbal and written lifestyle information would be preferable to only verbal advice, as patients receive too much information during their cancer journey and they cannot take in everything. This was viewed as preferable to written advice alone as it could be made more relevant to the patient. HPs felt that all HPs should provide lifestyle advice.

> *If I just gave them a leaflet… I think it needs to be given in conjunction with a health professional or somebody who can talk them through it. If you just give them another leaflet, they're just likely to put it with their pack of other things they've got.*
>
> Physiotherapist, F, 51y

HPs in favour of advice using a face-to-face meeting with a HP felt that this meeting should be part of the general follow-up to boost attendance and acceptability. They mentioned that the meeting could enhance continuity of care post-treatment,

For peer review only - http://bmjopen.bmj.com/site/about/guidelines.xhtml

fostering personal contact with a professional aware of the patient's journey. For the survivors who would prefer the support of a group-based intervention, local community venues were preferred over hospital rooms. Online advice was deemed appropriate for technology-literate survivors.

> *A group intervention is definitely a positive thing for patients to get that peer support from other patients as well. I think it helps patients to feel that they can manage their condition better themselves and also with support from other patients rather than it just being medically led all the time, particularly if it's off site as well, if it's in other, perhaps, community settings as well.*
>
> Breast Cancer Nurse, F, 46y

> *I think online is probably the best thing, if patients have access and they're computer literate, because they can do it at their own time and at their own pace.*
>
> Colorectal Cancer Nurse, F, 55y

3.2 Small and achievable changes framed as part of their treatment regimen

HPs mentioned that survivors who need to change multiple behaviours might be overwhelmed by the process and suggested prioritising and tackling one or two behaviours at a time focusing on small realistic changes.

> *I'm aware that if you give people too much advice all at once, it's quite difficult for them to take it all in, so sometimes you are better off just focusing on one thing […] finding out which you think is the most risky of their behaviours and concentrate on that one.*
>
> Prostate Cancer Nurse, F, 53y

Some thought that framing the healthy lifestyle advice as part of their treatment would make it more engaging.

> *It would be nice to have some guidelines available, some specific booklet available about benefits of healthy diet and exercise, so we can give it to them as well, so they can follow it as a specific treatment regimen. [...] It is a treatment – healthy diet and exercise – and say it's a treatment. We should make them feel like that.*
>
> Breast Cancer Surgeon, M, 44y

For peer review only - http://bmjopen.bmj.com/site/about/guidelines.xhtml

> *I'd start telling them from the moment I see them with their diagnosis, because I want them to feel it's as important for them to undertake physical activity and eat a good diet and lose some weight as is their other treatments, like radiotherapy, and hormone blocking treatment. […] I tell them it will be part of their treatment so that they are prepared and accept it as normal.*
>
> Breast Cancer Surgeon, F, 41y

3.3 Cost-effectiveness for wide-scale implementation

Time and financial resources and proven cost-effectiveness were cited as barriers for wide-scale implementation of lifestyle advice. Lower need for resources indicated higher potential for implementation but HPs were unsure of measurable outcomes for one-off low-intensity lifestyle interventions, such as a leaflet or a phone call.

> *I think the patients would like the face-to-face. I suppose it just depends on what the resources are, as to who is doing it – is it a dietitian, is it a CNS, is it another organisation? So I think it depends on the manpower, what resources are available for a face-to-face. I think if it was extra on top of what we currently provide then we would struggle.*
>
> Colorectal Cancer Nurse, F, 54y

> *Integration is fine [for leaflet and online advice]. The question is: will it help?*
>
> Prostate Cancer Physician, F, 54y

Discussion

The current study reiterated that HPs' desire to provide lifestyle advice is not necessarily matched by knowledge and action. Reported barriers to provision included perceptions about survivor's behaviours, abilities and interest in advice, as well as concerns about HP's own abilities to provide effective advice without damaging their patient relationships. Nonetheless, HPs in this study were keen for most survivors to receive lifestyle advice throughout the cancer journey. Suggestions for this advice, included tailoring to individual patient needs and preferences, initiating small and achievable change goals, and framing recommendations as part of the patient's treatment regimen.

For peer review only - http://bmjopen.bmj.com/site/about/guidelines.xhtml

Previously reported barriers to provision of lifestyle advice include perception that patients lack interest in behaviour change, perception that behaviour change is not feasible or effective, lack of guidelines and confusion about the research findings, self-identification as not the right person to provide advice, potential for blaming the patient, lack of appropriate behaviour change support, and lack of time.[16 20 21] The current study confirmed the above barriers offering further insight, such as the perception that providing advice on alcohol and smoking is more challenging compared to diet and physical activity. Another insight was that the fear of blaming relates not only to cancer onset but also to cancer recurrence. The study also added to the list of barriers the potential loss of credibility and connection with survivors, the obstruction of free will, the importance of role modelling, and the challenge of addressing the co-existence of multiple suboptimal behaviours given practical barriers to implementing advice, such as affordability of a healthy diet. These factors could be taken into consideration in the development of programmes aiming to train HPs to effectively deliver lifestyle advice, as evidence suggests that even brief advice from HPs is effective for lifestyle behaviour change.[22-24]

Interestingly, the HPs' self-identification as not the right person to provide advice and their fear of losing the connection with their patients by providing them with continuous lifestyle advice, are not matched by the fact that survivors would actually welcome lifestyle advice from their oncology HPs.[13-15] Survivors' views partly support the perception of HPs that it is difficult to change engrained unhealthy lifelong habits. However, although there is little evidence that survivors make major changes in their health behaviours following a cancer diagnosis,[8 12] some report motivation to engage in behaviour change.[13 25] HP's therefore have an opportunity to capitalise on this and to provide lifestyle advice to survivors or signpost them to further support. These interventions should include social support, because this behaviour change technique was perceived to facilitate performing health behaviours by both HPs in the current study and survivors.[15]

However, HPs in this study did not mention reputable and long-standing lifestyle guidelines for cancer survivors.[11] One reason for the lack of awareness might be that these guidelines are based on the cancer prevention literature. However, the evidence base for their implementation post-diagnosis is growing, and they are

For peer review only - http://bmjopen.bmj.com/site/about/guidelines.xhtml

484 considered to be prudent advice for cancer survivors. The lack of dissemination of these guidelines should therefore be targeted with educational courses, conferences, and, training to enable HPs to provide evidence-based advice. Professional organisations might be pivotal in this process. Programmes to enhance oncology HPs knowledge on lifestyle guidelines and facilitate provision of advice are currently being piloted, and are showing promising potential.[26 27] These programmes should ensure not only adequate knowledge of the guidelines but also provide the tools and techniques to HPs to ensure they approach the topic of behaviour change in a supportive way with confidence and sensitivity, so that cancer survivors feel motivated and not guilty.

HPs in this study also indicated that the format of lifestyle advice should be tailored to individual survivors. Surveys, qualitative studies, and analysis of data from large behaviour change interventions are required to identify factors that predict cancer survivors' uptake and adherence to various types of lifestyle interventions. However, an analysis of three large home-based lifestyle interventions in cancer survivors did not identify any predictors of adherence to the different types of intervention.[28] To that end, quasi-experimental intervention designs (e.g. face-to-face vs. remote intervention) based on survivors' preferences for delivery format might better facilitate understanding of adherence and lead to effective personalised lifestyle support. Framing the intervention as part of the treatment regimen instead of health promotion advice could also be explored in future studies, with pilot studies indicating the effectiveness of physical activity rehabilitation on fitness levels.[29]

Strengths of the current study include the examination of HPs' views on key modifiable lifestyle behaviours from a sample of various HPs from across the UK. Although qualitative studies provide in-depth information, their findings cannot be generalised. It is possible that the participants of this study were generally interested in lifestyle topics, which might bias the responses towards a positive view on lifestyle. Furthermore, the views may not be representative of all oncology HPs. Further research could explore this, as HPs working with other cancers may be even less likely to give advice.

For peer review only - http://bmjopen.bmj.com/site/about/guidelines.xhtml

In conclusion, the findings of this study suggest that some, but not all, HPs specialising in breast, prostate and colorectal cancers provide lifestyle advice to their patients post-treatment. Those that provide advice describe providing generic guidance broadly based on healthy lifestyle recommendations for the general population, with attempts to tailor it to the individual patient. Additional education is needed to improve awareness of available guidelines for cancer survivors. Incorporation of the identified factors that influence the provision of lifestyle advice in the development of training programmes for HPs and lifestyle interventions for cancer survivors could increase the probability of effectiveness and in turn, improve outcomes for cancer survivors.

For peer review only - http://bmjopen.bmj.com/site/about/guidelines.xhtml

Acknowledgements

All authors were supported by funding from Cancer Research UK. SL was also funded by University College London and the Weight Concern Impact Award Studentship. RJB is currently supported by Yorkshire Cancer Research Academic Fellowship funding. The researchers are grateful to the health professionals that participated in the study.

Declaration of Conflicting Interests

The authors declare no conflict of interests.

Contributorship statement

RJB conceived the study and obtained the funding for the Impact Award Studentship. SL, RJB, AF, KW, and HC devised the interview protocol. SL conducted the interviews. SL, DAK, and RJB analysed the data with contribution from all authors. DAK drafted the manuscript. All authors critically revised, and approved the final version of the manuscript.

Data sharing

Anonymised interview transcripts can be obtained by the corresponding author on reasonable request.

For peer review only

References

1. Maddams J, Utley M, Moller H. Projections of cancer prevalence in the United Kingdom, 2010-2040. *Br J Cancer* 2012;107(7):1195-202. doi: 10.1038/bjc.2012.366
2. Quaresma M, Coleman MP, Rachet B. 40-year trends in an index of survival for all cancers combined and survival adjusted for age and sex for each cancer in England and Wales, 1971-2011: a population-based study. *Lancet* 2015;385(9974):1206-18. doi: 10.1016/S0140-6736(14)61396-9 [published Online First: 2014/12/07]
3. Keats MR, Cui Y, Grandy SA, et al. Cardiovascular disease and physical activity in adult cancer survivors: a nested, retrospective study from the Atlantic PATH cohort. *J Cancer Surviv* 2017;11(2):264-73. doi: 10.1007/s11764-016-0584-x [published Online First: 2016/11/18]
4. Duan W, Li S, Meng X, et al. Smoking and survival of breast cancer patients: A meta-analysis of cohort studies. *Breast* 2017;33:117-24. doi: 10.1016/j.breast.2017.03.012 [published Online First: 2017/04/04]
5. Schwedhelm C, Boeing H, Hoffmann G, et al. Effect of diet on mortality and cancer recurrence among cancer survivors: a systematic review and meta-analysis of cohort studies. *Nutr Rev* 2016;74(12):737-48. doi: 10.1093/nutrit/nuw045 [published Online First: 2016/11/20]
6. Stacey FG, James EL, Chapman K, et al. A systematic review and meta-analysis of social cognitive theory-based physical activity and/or nutrition behavior change interventions for cancer survivors. *J Cancer Surviv* 2015;9(2):305-38. doi: 10.1007/s11764-014-0413-z
7. Stolley M, Sheean P, Gerber B, et al. Efficacy of a Weight Loss Intervention for African American Breast Cancer Survivors. *J Clin Oncol* 2017:JCO2016719856. doi: 10.1200/JCO.2016.71.9856 [published Online First: 2017/06/20]
8. Rock CL, Doyle C, Demark-Wahnefried W, et al. Nutrition and physical activity guidelines for cancer survivors. *CA Cancer J Clin* 2012;62(4):243-74. doi: 10.3322/caac.21142
9. Schmitz KH, Courneya KS, Matthews C, et al. American College of Sports Medicine roundtable on exercise guidelines for cancer survivors. *Med Sci Sports Exerc* 2010;42(7):1409-26. doi: 10.1249/MSS.0b013e3181e0c112
10. WCRF. Continuous Update Project Report: Diet, Nutrition, Physical Activity, and Breast Cancer Survivors: World Cancer Research Fund International, 2014.
11. WCRF. Food, Nutrition, Physical Activity and the Prevention of Cancer: A Global Perspective. Washington, DC: World Cancer Research Fund / American Institute for Cancer Research, 2007.
12. Williams K, Steptoe A, Wardle J. Is a cancer diagnosis a trigger for health behaviour change? Findings from a prospective, population-based study. *Br J Cancer* 2013;108(11):2407-12. doi: 10.1038/bjc.2013.254
13. Beeken RJ, Williams K, Wardle J, et al. "What about diet?" A qualitative study of cancer survivors' views on diet and cancer and their sources of information. *Eur J Cancer Care (Engl)* 2016;25(5):774-83. doi: 10.1111/ecc.12529
14. Koutoukidis DA, Beeken RJ, Lopes S, et al. Attitudes, challenges, and needs about diet and physical activity in endometrial cancer survivors: a qualitative study. *Eur J Cancer Care (Engl)* 2016 doi: 10.1111/ecc.12531

For peer review only - http://bmjopen.bmj.com/site/about/guidelines.xhtml

15. Smith L, Croker H, Fisher A, et al. Cancer survivors' attitudes towards and knowledge of physical activity, sources of information, and barriers and facilitators of engagement: A qualitative study. *Eur J Cancer Care (Engl)* 2017;26(4) doi: 10.1111/ecc.12641 [published Online First: 2017/01/31]

16. Williams K, Beeken RJ, Fisher A, et al. Health professionals' provision of lifestyle advice in the oncology context in the United Kingdom. *Eur J Cancer Care (Engl)* 2015;24(4):522-30. doi: 10.1111/ecc.12305

17. Bengtsson M. How to plan and perform a qualitative study using content analysis. *NursingPlus Open* 2016;2(Supplement C):8-14. doi: https://doi.org/10.1016/j.npls.2016.01.001

18. Hsieh HF, Shannon SE. Three approaches to qualitative content analysis. *Qual Health Res* 2005;15(9):1277-88. doi: 10.1177/1049732305276687

19. Tong A, Sainsbury P, Craig J. Consolidated criteria for reporting qualitative research (COREQ): a 32-item checklist for interviews and focus groups. *Int J Qual Health Care* 2007;19(6):349-57. doi: 10.1093/intqhc/mzm042

20. Miles A, Simon A, Wardle J. Answering patient questions about the role lifestyle factors play in cancer onset and recurrence: what do health care professionals say? *J Health Psychol* 2010;15(2):291-8. doi: 10.1177/1359105309351245

21. Sutton E, Hackshaw-McGeagh LE, Aning J, et al. The provision of dietary and physical activity advice for men diagnosed with prostate cancer: a qualitative study of the experiences and views of health care professionals, patients and partners. *Cancer causes & control : CCC* 2017;28(4):319-29. doi: 10.1007/s10552-017-0861-7

22. NICE. Physical activity: brief advice for adults in primary care. In: Excellence NIfHaC, ed., 2013.

23. Jackson SE, Wardle J, Johnson F, et al. The impact of a health professional recommendation on weight loss attempts in overweight and obese British adults: a cross-sectional analysis. *BMJ Open* 2013;3(11):e003693. doi: 10.1136/bmjopen-2013-003693

24. Fisher A, Williams K, Beeken R, et al. Recall of physical activity advice was associated with higher levels of physical activity in colorectal cancer patients. *BMJ Open* 2015;5(4):e006853. doi: 10.1136/bmjopen-2014-006853

25. Anderson AS, Steele R, Coyle J. Lifestyle issues for colorectal cancer survivors--perceived needs, beliefs and opportunities. *Support Care Cancer* 2013;21(1):35-42. doi: 10.1007/s00520-012-1487-7

26. Webb J, Hall J, Hall K, et al. Increasing the frequency of physical activity very brief advice by nurses to cancer patients. A mixed methods feasibility study of a training intervention. *Public health* 2016;139:121-33. doi: 10.1016/j.puhe.2016.05.015

27. Murphy J, Worswick L, Pulman A, et al. Translating research into practice: evaluation of an e-learning resource for health care professionals to provide nutrition advice and support for cancer survivors. *Nurse Educ Today* 2015;35(1):271-6. doi: 10.1016/j.nedt.2014.05.009 [published Online First: 2014/06/28]

28. Adams RN, Mosher CE, Blair CK, et al. Cancer survivors' uptake and adherence in diet and exercise intervention trials: an integrative data analysis. *Cancer* 2015;121(1):77-83. doi: 10.1002/cncr.28978

29. West MA, Loughney L, Lythgoe D, et al. Effect of prehabilitation on objectively measured physical fitness after neoadjuvant treatment in preoperative rectal

For peer review only - http://bmjopen.bmj.com/site/about/guidelines.xhtml

639 cancer patients: a blinded interventional pilot study. *British journal of*
640 *anaesthesia* 2015;114(2):244-51. doi: 10.1093/bja/aeu318
641

For peer review only

Supplementary material 1: Interview Topic Guide

	Topic	Question	Prompt
1.	Introductions / background	Brief introductions; get verbal consent to tape-record the interview; remind the aims of study, check length of interview/audio-recorded.	
2.	Health beliefs	What are your views on the role of diet and physical activity in the recovery from [breast/prostate/colorectal] cancer?<br>What are your views on the role of diet and physical activity in cancer recurrence?	
3.	Awareness of lifestyle guidelines for cancer survivors	Are you familiar with any guidelines specifically for cancer patients for any of the following lifestyle topics?<br>You said that you were aware of "X, Y, Z" guidelines. Can you please tell me what you know (or what you remember) about each of these guidelines?	Prompt<br>- PA<br>- Diet<br>- Weight management<br>- Smoking<br>- Alcohol
		What are your views on these guidelines? What do you think about them?	Prompt<br>- Liking<br>- Realistic<br>- Reliable
		How did you find out about them?<br>You also said that you were not aware of the "K, M" guidelines. Why do you think that you did not come across these guidelines?<br>What were the barriers for you not getting this information?	
3.	Barriers for provision of lifestyle advice	Thinking of all your patients who have completed primary treatment for cancer, do you give your patients advice on any lifestyle topics?	Prompt<br>- PA<br>- Diet<br>- Weight management<br>- Smoking<br>- Alcohol
		You said that you give advice on "X lifestyle topic" to "X%" of your patients. What kind of advice do you tend to give?	Prompt<br>- Verbal

For peer review only

Topic	Question	Prompt
		- Written<br>- Website referral<br>- HP referral<br>- When / Why this specific format<br>- Guidelines based
	Why do you think that you provide advice on this to [so many / only to x%] of your patients?<br>What prevents you from providing this type of advice to more patients?<br>What are the reasons why you don't usually provide lifestyle advice?	Prompt<br>- Verbal<br>- Written<br>- Website referral<br>- HP referral
	For each lifestyle topic that professional <u>does NOT provide any advice</u>, ask: Is there any particular reason why you have not given advice on "X lifestyle topic" to any of your patients?<br>What are the barriers preventing you to provide this advice?	Prompt<br>- Guidelines
	On the survey, we asked you about a number of specific barriers preventing you to provide lifestyle advice for your patients. You mentioned that "X" and "Y" were the main barriers for you. Can you tell me more about these barriers? Why these prevent you from providing lifestyle advice?<br>Are there any other barriers?	
5. Content Expectations	Have any of your cancer patients ask you information about lifestyle?<br><br>What type of information do they usually ask for?<br>How often do they ask you about lifestyle information? Why?<br>Is there any other type of information they should be getting? Why do you think it is important they get that information?<br>Is there anything you would <u>avoid</u> in telling them? Why?	

For peer review only

Topic	Question	Prompt
	Have any of the relatives, family members, friends or carers of your patients asked you information about lifestyle?<br>What type of information do they usually ask for?<br>How often do they ask you about lifestyle information? Why?<br>Is there any other type of information they should be getting? Why do you think it is important they get that information?<br>Is there anything you would avoid in telling relatives, friends or carers? Why?	
6. Delivery	We are planning to develop some materials to provide information on diet and physical activity for people who have had cancer treatment.<br>When would it be the best time for health professionals to provide information on healthy eating and physical activity to colorectal cancer patients? Why?<br>Do you think patients would be open to receive this type of information at that time? Why?<br>Who should provide them information about healthy eating and physical activity? Why?<br>In what format do you think that it would be better to provide information about diet and physical activity, for colorectal cancer patients? Why?	
	What do you think about a leaflet?<br>What do you think about a leaflet along with a telephone call from a HP?<br>What do you think about a leaflet along with a face-to-face contact with a HP?<br>What do you think about a leaflet along with online information?<br>What do you think about a self-help book with specific information for cancer survivors?<br>What do you think about a group intervention designed for cancer survivors with about 8 sessions, providing more detailed lifestyle information?<br>We have discussed about several options and formats to provide lifestyle information. Balancing between what would be better for HPs, for patients and feasibility of implementation; what do you think would be the best format to provide lifestyle advice?	Prompt<br>- Usefulness<br>- Receipt<br>- Implementation

For peer review only

	Topic	Question	Prompt
		From your perspective, what are the issues or barriers to take into account that some [breast/prostate/colorectal] cancer patients might face in trying to change their lifestyle?	Prompt<br>- Mood<br>- Mental health<br>- Physical restrictions<br>- Visual problems
7.	Ending	Do you have anything else to add, any comments or any questions?	

For peer review only

Supplementary material 2 – Interviews NVIVO Coding Framework

(1) Health Beliefs

- Beliefs about role of diet in cancer (incl. cancer recurrence)
- Beliefs about role of exercise in cancer (incl. cancer recurrence)
- Beliefs about role of alcohol in cancer (incl. cancer recurrence)
- Beliefs about role of smoking in cancer (incl. cancer recurrence)
- Health beliefs (not lifestyle)

(2) Awareness of lifestyle guidelines for cancer survivors

- Aware of guidelines (& description)
- Not aware of guidelines (& description)
- Views on guidelines
- Barriers to accessing guidelines (incl. not aware)
- How found out about guidelines

(3) Provision of lifestyle advice

- Advice given (diet)
- Advice given (exercise)
- Advice given (smoking)
- Advice given (alcohol)
- Advice given (weight)
- Advice given and requested by Relatives
- Advice would like to get on other topics
- Barriers to provision of advice
- Reason for provision of advice to a HIGH number of pts
- Reason for provision of advice to a LOW number of pts

(4) Format

- Timing
    - Timing HP has provided lifestyle advice
    - Preferred timing for provision of lifestyle advice
- Source

For peer review only

Mode of delivery (all negative, incl. leaflet, self-help book and group)

Ideal Mode of delivery

Mode of delivery of information provided (incl verbally, referral, web, leaflet, self help and group)

Format Group

Format Verbally

Format Digital

Format Written (All)

Leaflet only

Leaflet + telephone call

Leaflet + face-to-face contact

Leaflet + online information

Self-Help book

(5) Barriers to attending lifestyle interventions - internal & external

(6) Pathway issues

Leaflet only

Leaflet + telephone call

Leaflet + face to face contact

Leaflet + online information

Self-Help Book

Group intervention

Other Pathway factors

For peer review only

COREQ (COnsolidated criteria for REporting Qualitative research) Checklist

A checklist of items that should be included in reports of qualitative research. You must report the page number in your manuscript where you consider each of the items listed in this checklist. If you have not included this information, either revise your manuscript accordingly before submitting or note N/A.

Topic	Item No.	Guide Questions/Description	Reported on Page No.
**Domain 1: Research team and reflexivity**			
Personal characteristics			
Interviewer/facilitator	1	Which author/s conducted the interview or focus group?	5
Credentials	2	What were the researcher's credentials? E.g. PhD, MD	5
Occupation	3	What was their occupation at the time of the study?	5
Gender	4	Was the researcher male or female?	5
Experience and training	5	What experience or training did the researcher have?	5
Relationship with participants			
Relationship established	6	Was a relationship established prior to study commencement?	5
Participant knowledge of the interviewer	7	What did the participants know about the researcher? e.g. personal goals, reasons for doing the research	5
Interviewer characteristics	8	What characteristics were reported about the inter viewer/facilitator? e.g. Bias, assumptions, reasons and interests in the research topic	5
**Domain 2: Study design**			
Theoretical framework			
Methodological orientation and Theory	9	What methodological orientation was stated to underpin the study? e.g. grounded theory, discourse analysis, ethnography, phenomenology, content analysis	5
Participant selection			
Sampling	10	How were participants selected? e.g. purposive, convenience, consecutive, snowball	5
Method of approach	11	How were participants approached? e.g. face-to-face, telephone, mail, email	5
Sample size	12	How many participants were in the study?	5-6
Non-participation	13	How many people refused to participate or dropped out? Reasons?	5-6
Setting			
Setting of data collection	14	Where was the data collected? e.g. home, clinic, workplace	5
Presence of non-participants	15	Was anyone else present besides the participants and researchers?	N/A
Description of sample	16	What are the important characteristics of the sample? e.g. demographic data, date	6-7
Data collection			
Interview guide	17	Were questions, prompts, guides provided by the authors? Was it pilot tested?	5
Repeat interviews	18	Were repeat inter views carried out? If yes, how many?	N/A
Audio/visual recording	19	Did the research use audio or visual recording to collect the data?	5
Field notes	20	Were field notes made during and/or after the inter view or focus group?	5
Duration	21	What was the duration of the inter views or focus group?	5
Data saturation	22	Was data saturation discussed?	N/A
Transcripts returned	23	Were transcripts returned to participants for comment and/or	N/A

For peer review only

Topic	Item No.	Guide Questions/Description	Reported on Page No.
		correction?	
**Domain 3: analysis and findings**			
Data analysis			
Number of data coders	24	How many data coders coded the data?	5
Description of the coding tree	25	Did authors provide a description of the coding tree?	Appendix 2
Derivation of themes	26	Were themes identified in advance or derived from the data?	5-6
Software	27	What software, if applicable, was used to manage the data?	5
Participant checking	28	Did participants provide feedback on the findings?	N/A
Reporting			
Quotations presented	29	Were participant quotations presented to illustrate the themes/findings? Was each quotation identified? e.g. participant number	7-18
Data and findings consistent	30	Was there consistency between the data presented and the findings?	7-18
Clarity of major themes	31	Were major themes clearly presented in the findings?	7-18
Clarity of minor themes	32	Is there a description of diverse cases or discussion of minor themes?	7-18

Developed from: Tong A, Sainsbury P, Craig J. Consolidated criteria for reporting qualitative research (COREQ): a 32-item checklist for interviews and focus groups. *International Journal for Quality in Health Care*. 2007. Volume 19, Number 6: pp. 349 – 357

Once you have completed this checklist, please save a copy and upload it as part of your submission. DO NOT include this checklist as part of the main manuscript document. It must be uploaded as a separate file.

For peer review only
